# Lipid Body Dynamics in Shoot Meristems: Production, Enlargement, and Putative Organellar Interactions and Plasmodesmal Targeting

**DOI:** 10.3389/fpls.2021.674031

**Published:** 2021-07-21

**Authors:** Manikandan Veerabagu, Päivi L. H. Rinne, Morten Skaugen, Laju K. Paul, Christiaan van der Schoot

**Affiliations:** ^1^Faculty of Biosciences, Department of Plant Sciences, Norwegian University of Life Sciences, Ås, Norway; ^2^Faculty of Chemistry, Biotechnology, and Food Science, Norwegian University of Life Sciences, Ås, Norway

**Keywords:** LB/LD proteome, oleosin, LDIP, LDAP, Caleosin, ACT7, Myosin XI-binding Rab C2A, plasmodesmata

## Abstract

Post-embryonic cells contain minute lipid bodies (LBs) that are transient, mobile, engage in organellar interactions, and target plasmodesmata (PD). While LBs can deliver γ-clade 1,3-β-glucanases to PD, the nature of other cargo is elusive. To gain insight into the poorly understood role of LBs in meristems, we investigated their dynamics by microscopy, gene expression analyzes, and proteomics. In developing buds, meristems accumulated LBs, upregulated several LB-specific *OLEOSIN* genes and produced OLEOSINs. During bud maturation, the major gene *OLE6* was strongly downregulated, OLEOSINs disappeared from bud extracts, whereas lipid biosynthesis genes were upregulated, and LBs were enlarged. Proteomic analyses of the LB fraction of dormant buds confirmed that OLEOSINs were no longer present. Instead, we identified the LB-associated proteins CALEOSIN (CLO1), Oil Body Lipase 1 (OBL1), Lipid Droplet Interacting Protein (LDIP), Lipid Droplet Associated Protein1a/b (LDAP1a/b) and LDAP3a/b, and crucial components of the OLEOSIN-deubiquitinating and degradation machinery, such as PUX10 and CDC48A. All mRFP-tagged LDAPs localized to LBs when transiently expressed in *Nicotiana benthamiana*. Together with gene expression analyzes, this suggests that during bud maturation, OLEOSINs were replaced by LDIP/LDAPs at enlarging LBs. The LB fraction contained the meristem-related actin7 (ACT7), “myosin XI tail-binding” RAB GTPase C2A, an LB/PD-associated γ-clade 1,3-β-glucanase, and various organelle- and/or PD-localized proteins. The results are congruent with a model in which LBs, motorized by myosin XI-k/1/2, traffic on F-actin, transiently interact with other organelles, and deliver a diverse cargo to PD.

## Introduction

Plasmodesmata (PD) are notoriously difficult to investigate, and understanding PD functioning from the composition is fraught with difficulties. Both composition and architecture of PD are subject to regulation by cells that share them. Rather than functioning autonomously, they are subject to control by cells that construct, maintain, and operate them. Consequently, PD composition and function are context-dependent, differing between tissues in dependence on developmental and metabolic cellular states. Filtering out commonalities while recognizing the unique aspects of PD at specific locations and conditions, therefore, remains a challenge.

Information on PD composition and function has been gathered using a variety of plant and tissue systems and approaches. Frequently, investigations are focused on PD (ultra)structure and/or localization of suspected PD proteins by immunochemistry or transgenic expression of fluorescently tagged proteins, microinjection studies, and proteomic and lipidomic studies (reviewed in Faulkner and Maule, 2011; Sager and Lee, 2014; Heinlein, 2015a; Brault et al., 2019; Han et al., 2019; Reagan and Burch-Smith, 2020).

Despite the importance of determining PD composition and architecture, the question remains unanswered of how relevant PD components are delivered to PD and integrated into the functional fabric of the PD channel. The cellular mechanisms that deliver structural PD components also modulate gating events. While the exterior and interior of PD are targeted by distinct mechanisms, PD conductance is also subject to control by physiological processes on both sides of the channels. For example, nuclear-organellar signaling contributes to ROS-mediated plasmodesmal regulation, involving mitochondria and chloroplasts as sensors for cellular homeostasis (Burch-Smith and Zambryski, 2011).

Regarding supply routes, the most frequently studied route is the Brefeldin-sensitive excretion pathway, which delivers proteins to the cell wall (Sager and Lee, 2014; Han et al., 2019; Reagan and Burch-Smith, 2020). Cargos delivered *via* this pathway may include integral membrane proteins and GPI-anchored proteins. The latter can attach to exoplasmic membrane rafts, which move them laterally to the exterior of the PD (Mongrand et al., 2010). With regard to the PD interior, important leads emerged from studies that analyzed how viruses take advantage of existing cellular mechanisms, such as the cortical endoplasmic reticulum (ER) and the cytoskeleton, and how they interact with molecular complexes that control PD gating (reviewed in e.g., Heinlein, 2015b; Reagan and Burch-Smith, 2020). Little explored is the mechanism by which cytoplasmically produced lipid bodies (LBs) target PD to deliver a largely unknown cargo to the channel (Rinne et al., 2011; Veerabagu et al., 2020).

In seeds, LB production requires OLEOSINs (Huang, 1992; Tzen and Huang, 1992; Abell et al., 2002). OLEOSIN proteins are small 15–26 kD proteins that are co-translationally inserted into the bilayer of the ER, guided by an ER-resident signal recognition particle (Abell et al., 2004). The 5–6 nm long hydrophobic hairpin of OLEOSIN is embedded under strain in the bilayer, which facilitates its diffusion into the stable hydrophobic environment of a nascent LB, promoting its eventual release into the cytosol (Abell et al., 2002; Huang and Huang, 2017; Huang, 2018). This budding process is facilitated by a critical imbalance in leaflet surface tensions and involves SEIPIN proteins and interacting lipid biosynthesis genes (Cai et al., 2015; Barbosa and Siniossoglou, 2017). Once formed, OLEOSIN secures the integrity and small size of the LBs by stabilizing the monolayer and preventing coalescence and fusion (Siloto et al., 2006; Shimada et al., 2008; Hsiao and Tzen, 2011). In addition, CALEOSIN and STEROLEOSIN can bind competitively with an expanding monolayer, mediated by short ca. 2 nm hydrophobic hairpins (Huang, 2018).

Like seeds, bud meristems contain LBs, and eight of the nine *Populus OLEOSIN* genes are expressed in apices (Veerabagu et al., 2020). The capacity of LBs to deliver cargos to PD could be important in the shoot apical meristem (SAM), where PD are continuously produced within and between cell lineages (van der Schoot and Rinne, 1999). In the active SAM, individual cells are continuously displaced toward the periphery to be integrated into differentiating tissues. To secure the functional integrity of the SAM, all cells need to continuously update their relative position by exchanging signals, among others, through existing and newly formed PD (Rinne and van der Schoot, 1998). LBs potentially contribute to PD formation, maintenance, and cell-cell signaling by shuttling lipids, enzymes, and signaling molecules to the PD entrance (van der Schoot et al., 2011; Paul et al., 2014a). An LB shuttle function was demonstrated in *N. benthamiana*, where LBs delivered eGFP-tagged 1,3-β-glucanases to PD, identified by TMV MP-mRFP (Rinne et al., 2011). Similarly, transgenic Arabidopsis LBs, tagged with PtOLE6-eGFP, targeted primary and secondary PD in various cell types (Veerabagu et al., 2020). The LBs do not arrive at the PD by bulk cytoplasmic streaming but by processive trafficking on F-actin, mediated by myosin XI-k/1/2 (Veerabagu et al., 2020).

The PD in the SAM of woody perennials are unique in the sense that they are modified during the seasonal cycle. Under short days, the PD are shut down by Dormancy Sphincter Complexes (DSCs). DSCs act as circuit breakers that interrupt the symplasmic circuitry of the SAM, preventing electrical and metabolic coupling and exchange of transcription factors and other regulatory molecules, arresting the SAM in a dormant state (Paul et al., 2014a,b). Unlike classical sphincters where callose is present extracellularly, DSCs contain additional internal deposits that can be targeted by LBs (Rinne et al., 2001; Rinne and van der Schoot, 2003). When recruited to the PD, the LB-associated enzyme 1,3-β-glucanase aligns with its substrate, resulting in callose hydrolysis, restoration of the PD channel, and dormancy release (Rinne et al., 2001; Rinne and van der Schoot, 2003). It is unknown what other cargos LBs can deliver to PD during dormancy release, and to what degree it differs from what is present in active meristems. A consensus view is that cytoplasmic LB motility enriches LB cargos by facilitating organellar interactions and exchange of proteins and lipids (Bartz et al., 2007; Hodges and Wu, 2010; Murphy, 2012; Krahmer et al., 2013; Gao and Goodman, 2015; Zhi et al., 2017). Proteins might also be recruited directly from the cytoplasm, especially when molecular crowding at the monolayer is reduced. These include monolayer-embedded proteins, lipophilic signals, lipid-anchored proteins, electrostatically associated proteins, and molecules that opportunistically hitch a ride on moving LBs (reviewed in van der Schoot et al., 2011; Paul et al., 2014a; Huang, 2018).

As in buds, accumulated LBs constitute a unique proactive dormancy-release and signaling mechanism, LBs may be expected to store proteins related to these functions. LB cargos are likely to include proteins that assist docking at the plasma membrane (PM), possibly proteins that become integrated into the fabric of PD, and non-cell-autonomous signals (van der Schoot et al., 2011; Paul et al., 2014a,b). How the actomyosin system that guides LBs to PD (Veerabagu et al., 2020) connects to the PD is unknown. Notably, F-actin and myosin VIII have been localized at PD (White et al., 1994; Baluska et al., 2001; Golomb et al., 2008; White and Barton, 2011). In addition, the actin-binding and nucleation complex Arp2/3 was localized at PD (Van Gestel et al., 2003; Deeks and Hussey, 2005; Fiserova et al., 2006), and recently it has been shown that the class I formin FH2 acts as an actin nucleation factor that caps and stabilizes F-actin at PD (Diao et al., 2018). Some plant viruses that move through PD have usurped and hijacked the cytoplasmic actomyosin system to facilitate their cell-to-cell transport (Amari et al., 2011, 2014; Sager and Lee, 2014; Heinlein, 2015b). Although actin can facilitate delivery of viral complexes to the PD entrance, PD-associated actin might restrict the size of the PD channel as virus passage requires severing of this actin (Ding et al., 1996; Su et al., 2010).

While it is unknown how the F-actin on which LBs traffic is anchored to the PD, it is also unknown what enables LBs to dock at PD. Given the difference between LB and PD diameters in dormant meristems, respectively, ca. 1 μm and 60–220 nm (channel and external ring; Rinne and van der Schoot, 2004), it might involve proteins that interact with PD orifices or their immediate surroundings at the PM. Targeting of eGFP-tagged LBs to the PD/PM area yields deflated LBs that appear as juxtaposed fluorescent patches, sandwiching primary and secondary PD (Rinne et al., 2011; Veerabagu et al., 2020). We hypothesized earlier that LBs could dock at remorin-decorated membrane rafts that act as sorting devices (van der Schoot et al., 2011; Paul et al., 2014a) and involve hemifusion between the LB monolayer and the cytoplasmic leaflet of the PM. If so, this might be mediated by SNARE protein complexes, which can localize to LBs (Boström et al., 2007; Sollner, 2007; Murphy et al., 2009; Paul et al., 2014a).

The relation of LBs with PD is virtually unexplored. To gauge how LBs might contribute to cellular homeostasis, organellar interaction, and cargo delivery to PD, we investigated their accumulation and putative composition by microscopy, gene expression analyzes, and proteomics. An important goal was to create an inventory of candidate proteins, such as known LB proteins and proteins that may hitch a ride to the PD. The results indicate that OLEOSIN is responsible for LB accumulation but that at a later stage it is removed and replaced by LDIP/LDAP proteins to allow recruitment of cytoplasmic proteins through reduced molecular crowding at the monolayer. Removal and degradation of OLEOSIN probably involve the action of PUX10, the segregase CDC48A, and the 26S proteasome, all of which were present in the LB fraction. Other identified proteins included CLO1, OBL1, GPAT8, a PD/LB-localized 1,3-β-glucanase, and proteins that likely reflect LB motility, organellar interactions, storage, and docking to PD/PM sites. Confirming LB/PD localization and the role of individual proteins in PD functioning will require future investigations. The current data lay the groundwork for such investigations and expand a model (Veerabagu et al., 2020) in which cargo-enriched LBs are anchored by GTPase RAB-C2A to myosin XI-k/1/2 for actin-guided transport to PD.

## Results

### LBs in Apices and Developing Buds

**Figure 1:**
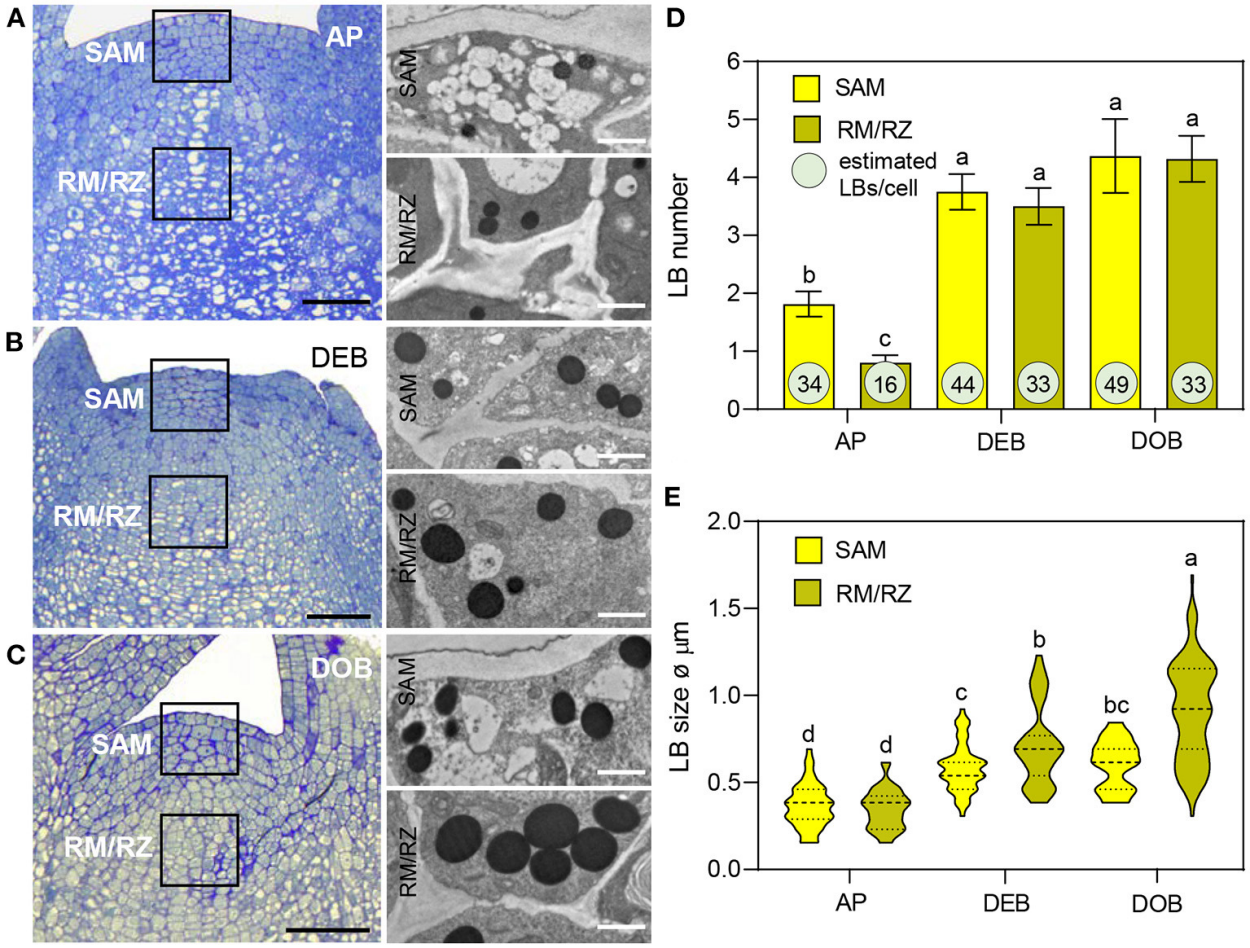
Lipid body (LB) production in the shoot apex of *Populus*. **(A–C)** show apices of **(A)** actively growing plants in long days (APs), **(B)** in short day-induced developing buds (DEBs) and **(C)** dormant buds (DOBs). TEM images **(A–C)** show LBs in the shoot apical meristem (SAM) and the rib meristem/rib zone (RM/RZ) (boxed meristem areas). **(D)** LB numbers were counted and **(E)** LB diameters were measured in successive TEM images. Bar diagram **(D)** shows means ± SD of LB numbers per cell per section. The encircled numbers are calculated LB numbers per cell volume based on average cell sizes, as explained in Supplementary Figure 1. **(E)** Violin plots show diameters of LBs including mean-line and quartiles. Different letters indicate statistically significant differences between treatments (one-way ANOVA, Fisher's *post-hoc* analysis *P* < 0.05). Bars are 50 (apices) and 1 μm (LBs).

Lipid bodies have been detected in meristems previously but their accumulation patterns have not been characterized. In this study, LB production, size, and number were analyzed from transmission electron microscope (TEM) sections of meristems of growing plants (APs), developing terminal buds (DEBs), and dormant terminal buds (DOBs) (Figures 1A–C). In all three, LBs were present in the SAM and the subjacent rib meristem/rib zone (RM/RZ) area but their numbers were low in actively growing apices, especially in the RM/RZ area (Figure 1D). The low number in this area reflects the high rate of metabolism and cell division related to stem elongation. During bud development, apical stem elongation ceases and LB numbers in the RM/RZ area and the SAM increased significantly, while further increase during dormancy development was minor (Figure 1D). Based on LB numbers detected in TEM sections, we approximated their total number for an average cell volume in both the SAM and RM/RZ. The calculation accounted for section thickness (80 nm), LB diameter, and the volume of the nucleus and organelles (Figure 1E, Supplementary Figure 1). These data showed that all cells contained multiple LBs, even the RM/RZ of actively growing long-day plants. In developing buds, the number of LBs in SAM cells had increased by ca. 25%, while in the RM/RZ it had increased by ca. 50%, corresponding to the cessation of cell division (Figure 1D), but beyond that, the increase in number was minor, and only in the SAM by ca. 10%. The increase in LB sizes showed a similar trend. In growing apices, LBs were small (Figures 1A,E), but their size had significantly increased in developing buds, both in the SAM and the RM/RZ (Figures 1B,E). During dormancy establishment, LB size only increased further in the RM/RZ (Figures 1C,E).

### OLEOSIN Expression and LB Enlargement

**Figure 2:**
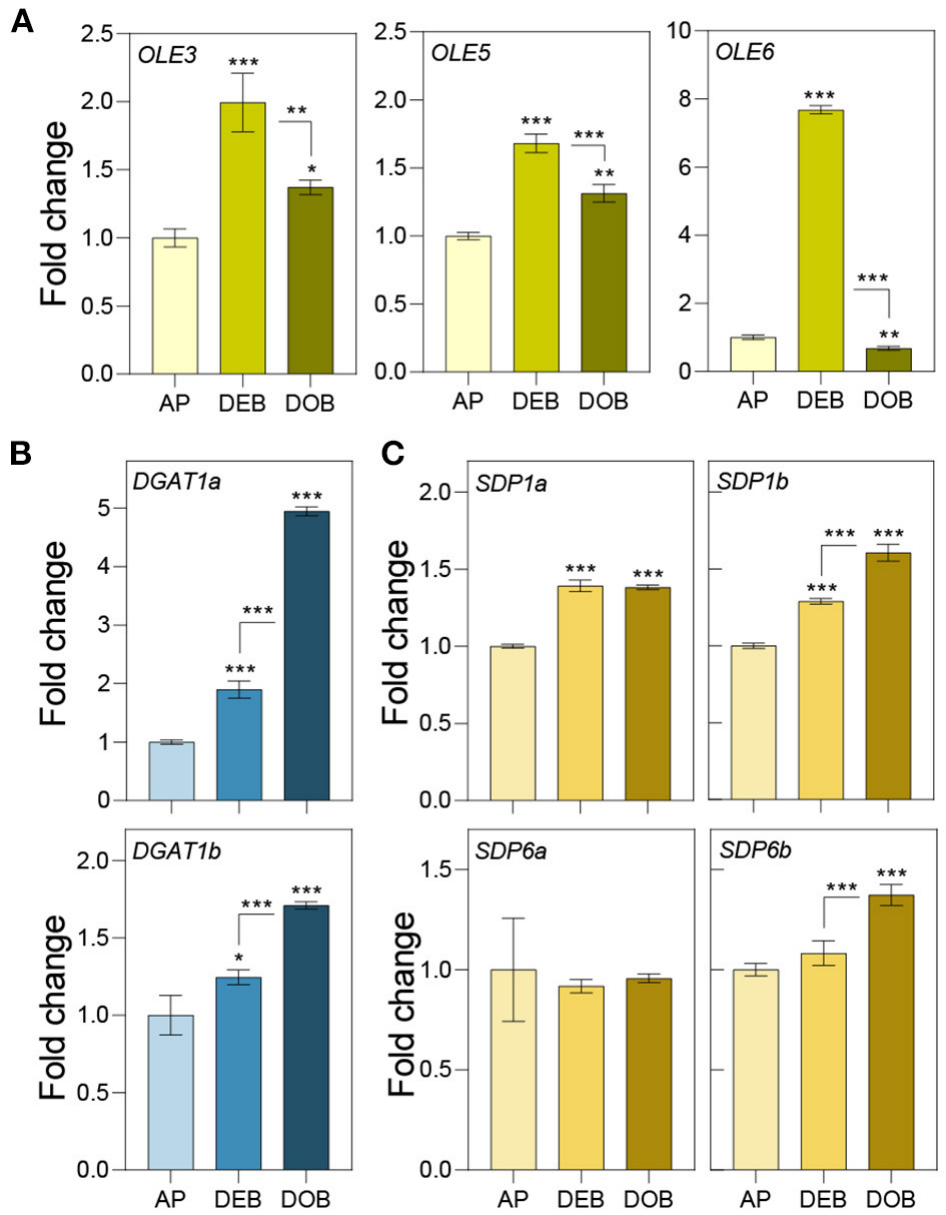
Expression of genes involved in lipid body production and lipid turnover. Relative expression of **(A)** *OLEOSIN* genes *(OLE3, OLE5*, and *OLE6)*, **(B)** lipid biosynthesis genes (*DGAT1a,b*), and **(C)** lipase genes (*SDP1a* and b, and *SDP6a* and *b*) in apices under long day (APs), and in developing buds (DEBs), and dormant buds (DOBs) in short days. Values are calculated relative to the control (AP) and represent the means of three technical replicates ± SD of four plants. Asterisk(s) indicate statistical significance between treatments (one-way ANOVA, Tukey analysis; *P < 0.05; **P < 0.01; and ***P < 0.001).

Previously, we showed that very early under dormancy-inducing conditions three of the eight expressed *OLEOSINs* were upregulated (Veerabagu et al., 2020). In this study, we analyzed their expression in growing apices, in developing buds during the LB accumulation phase (Figure 1D), and in buds that were developing dormancy (Figure 2A). *OLE6* appeared to be the most important of the three *OLEOSINs*, both quantitatively and in its responsiveness to bud development. It was strongly upregulated during the LB accumulation phase and almost completely downregulated during dormancy development. In contrast, the minor genes *OLE3* and *OLE5* were only slightly upregulated during LB production and somewhat downregulated during dormancy development (Figure 2A).

To investigate if LB enlargement (Figures 1C,E) could be due to increased TAG biosynthesis, which is mediated by DGAT1 (Shockey et al., 2006), we identified two *DGAT1* homologs, *DGAT1a* and *DGAT1b*, in the *Populus trichocarpa* genome (Supplementary Figure 2) and analyzed their expression during the same developmental stages. Both *DGAT1a* and *DGAT1b*, but especially *DGAT1a*, were upregulated during bud development and further during dormancy establishment (Figure 2B). This suggests that TAG biosynthesis may have increased concomitant with LB enlargement (Figure 1E). As the expression of lipases might counteract LB enlargement through TAG-hydrolysis, we also analyzed transcript levels of the lipase gene *SUGAR DEPENDENT 1* (*SDP1*) and the mitochondrial gene *SDP6*, which is required for post-germinative seedling growth in *Arabidopsis* (Quettier and Eastmond, 2009). Two isoforms of both *SDP1* and *SDP6* were identified in the *P. trichocarpa* genome (Supplementary Figure 3). *SDP1a* and *SDP1b* were upregulated during bud development, whereas *SDP1b* was further upregulated during dormancy establishment. The *SDP6a* expression did not show any change, and *SDP6b* was only slightly upregulated during dormancy establishment (Figure 2C). Considering that LBs enlarged, the encoded enzymes might not have targeted the LBs, like in seeds where *SPD1* transcript levels do not correlate with enzyme activity (Eastmond, 2006).

### LB-Associated Proteins in Dormant Buds

To identify integral LB proteins (referred to as class I) as well as proteins with amphipathic stretches (class II) and peripherally and transiently associated proteins (Bersuker and Olzmann, 2017; Pyc et al., 2017), we isolated and purified LBs from dormant buds using a protocol modified after Jolivet et al. (2004). Purified LBs were subjected to light and differential interference contrast (DIC) microscopy to confirm the absence of cellular fragments. Total fatty acid methyl ester (FAME) contents were quantified to verify the enrichment of fats in the final LB fraction (Supplementary Figure 4). LBs stained with the neutral lipid stain Nile red were further inspected under a confocal laser scanning microscope (CLSM) (Veerabagu et al., 2020). When visible contaminations were completely absent and all LBs were uniform and isodiametric in size, samples were prepared for protein precipitation, and extracted proteins were processed, trypsin-digested, and analyzed by liquid chromatography with tandem mass spectrometry (LC-MS/MS) as described in experimental procedures. MS/MS samples were analyzed using Mascot to search the *P. trichocarpa* database, whereas Scaffold was used to validate MS/MS-based peptide and protein identifications. We analyzed three independent pooled samples, obtained from different sets of plants that were grown at different times, representing biological replicates. To prevent loss of peripherally and transiently associated proteins that might be delivered to PD, such as, for example, the LB- and PD-localized γ-clade 1,3-β-glucanases, we deliberately omitted the commonly used salt step in the final wash of all three samples. However, the third sample received an extra wash, which diminished the number of identified proteins in the LB fraction. All proteins were identified with >99% probability, containing at least two unique peptides but often much more. In total, we identified 719 proteins in the LB fraction, of which many were still likely contaminants. To restrict the number of putative candidate proteins, we made a selection based on the previously reported presence of identified proteins at LBs or in LB fractions of plant and non-plant systems, localization at PD, or presence in PD-enriched fractions (Table 1).

**Table 1:** Proteins in the LB fraction, identical or similar to described putative LB/PD proteins.

**Protein name**	***P. trichocarpa***	**BlastP of**	**Mw**	**Unique peptides**		
	**Accession nr**	***A. thaliana***	**kDa**	**Bio1**	**Bio2**	**Bio3**
**Localized to plant LBs**						
LDAP Interacting Protein (LDIP)^1, 3, 8, 27, h^	Potri.004G082300	AT5G16550	24	3	3	4
LD-Associated Protein 3 (LDAP3a)^2, 4, 5, 6, 8^	Potri.005G025700	AT3G05500	27	11	11	4
LD-Associated Protein 3 (LDAP3b)^4, 5, 6, 8^	Potri.013G017300	AT3G05500	27	8	10	5
LD-Associated Protein 1 (LDAP1a)^3, 5, 6, 8, 27, 28^	Potri.003G173100	AT1G67360	25	9	9	8
LD-Associated Protein 1 (LDAP1b)^3, 5, 6, 8, 27, 28^	Potri.001G055300	AT1G67360	25	7	9	8
Oil body lipase 1 (OBL1a)^8, 28, 29^	Potri.001G161500	AT3G14360	65	5	8	11
Oil body lipase 1 (OBL1b)^8, 28, 29^	Potri.003G073800	AT3G14360	63	5	5	6
Caleosin (CLO1)^8^	Potri.010G066600	AT4G26740	27	4	4	2
Plant UBX-domain protein 10 (PUX10)^7, 8^	Potri.003G145200	AT4G10790	52	12	13	8
Cell division control protein 48A (CDC48A)^7, 8, 28^	Potri.012G088200	AT5G03340	90	56	62	7
Beta-1,3-endoglucanase (GH17-44)^9^	Potri.T167100	AT4G16260	35	3	3	4
GPAT8 (redundant with GPAT4)^31, 32^	Potri.014G085500	AT4G00400	56	3	8	5
**26S Proteasome**						
Regulatory particle AAA-ATPase 2A (RPT2A)	Potri.014G194700	AT4G29040	50	18	15	3
26S proteasome, regulatory subunit RPN7	Potri.015G090900	AT4G24820	45	8	8	2
Ubiquitin conjugating enzyme 13A (UBC13A)	Potri.011G111400	AT1G78870	18	4	5	2
Ubiquitin-activating enzyme E1 1 (UBA1)	Potri.009G075700	AT2G30110	121	27	23	6
**RAB GTPases—Myosin XI tail binding**						
Rab GTPase C2A (RAB-C2A/RAB18)^11, 14−16, 18, 23−26^	Potri.006G121400	AT5G03530	23	4	5	2
Rab GTPase D1 (RAB-D1)	Potri.003G004000	AT3G11730	23	8	7	4
**Actin/Microtubule**						
Actin 7 (ACT7)^27, b^	Potri.019G010400	AT5G09810	42	23	23	16
V-ATPase B Subunit 2 (VAB2; stabilizes F-actin)	Potri.009G137800	AT4G38510	54	25	24	21
Actin Depolymerizing Factor 4 (ADF4)	Potri.009G028200	AT5G59890	16	6	4	3
**PD/PM localized/PD enriched fraction**						
Purple acid phosphatase (PAP1)^b, h^	Potri.010G158400	AT1G13750	69	3	3	2
Calreticulin 1A (CRT1 /CRT1A)^b, h^	Potri.005G015100	AT1G56340	48	13	10	4
Calreticulin 1B (CRT2 / CRT1B)^b^	Potri.013G009500	AT1G09210	47	17	13	5
Calnexin 1 (CNX1)^23, 24, 25, 28, a, b^	Potri.012G111100	AT5G61790	61	29	28	14
Zerzaust (ZET), atypical β-1,3 glucanase^b, h^	Potri.019G032900	AT1G64760	53	4	4	2
O-Glycosyl hydrolases family 17 protein^b, h^	Potri.006G080600	AT5G58090	53	5	6	2
O-Glycosyl hydrolases family 17 protein^b, h^	Potri.018G150400	AT5G58090	52	9	8	3
β-1,3-glucanase 1 (BG1) (GH17_37/at PM)	Potri.016G057400	AT3G57270	37	4	5	3
Dehydrin (HIRD11)^c^	Potri.013G062200	AT1G54410	24	11	8	4
Early Responsive to Dehydration 4 (ERD4)^h^	Potri.001G358300	AT1G30360	82	9	5	5
Reticulon like protein B3 (RTN3)^18, b, f, g^	Potri.001G097700	AT1G64090	28	6	7	7
Reticulon like protein B1 (RTN1)^18, b^	Potri.015G027300	AT4G23630	30	3	2	3
Leucine-rich repeat (LRR) family protein^b^	Potri.001G017500	AT3G20820	40	12	13	7
Probable inactive receptor kinase^h^	Potri.018G074300	AT2G26730	71	9	3	2
Reversibly glycosylated polypeptide 3 (RGP3)^h^	Potri.010G156700	AT3G08900	41	36	32	11
Pectinacetylesterase 11 (PAE11/at PD)^TAIR^	Potri.004G233900	AT5G45280	43	14	16	5
Plasma membrane intrinsic protein 1 (PIP1)^h^	Potri.003G128600	AT4G00430	31	6	4	2
Plasma membrane intrinsic protein 3 (PIP3)^b^	Potri.009G136600	AT4G35100	30	4	3	2
Clathrin Heavy Chain 1 (CHC1)^14, 18^	Potri.009G073300	AT3G11130	193	55	63	38
SKU5 similar 1 (SKS1)^b, h^	Potri.015G127200	AT4G25240	54	3	2	2
**Plant LB fractions**						
SecY transport family protein/LB-targeting^28^	Potri.011G107900	AT2G34250	52	8	5	5
Annexin 1 (ANN1)^28, b^	Potri.002G095600	AT1G35720	36	23	22	16
Annexin 2 (ANN2)^20, 28^	Potri.005G075900	AT5G65020	36	25	23	9
Early responsive to dehydration 7 (ERD7)^a, 28^	Potri.004G174100	AT2G17840	48	12	9	11
Late embryogenesis abundant (LEA26)^28, b^	Potri.007G146300	AT2G44060	35	14	15	11
Late embryogenesis abundant protein (LEA27)^28^	Potri.002G165000	AT2G46140	16	6	4	4
Cytochrome P450 (CYP81K1)^28^	Potri.017G028100	AT5G10610	59	5	8	5
Cytochrome P450 (CYP94B1)^28^	Potri.005G220900	AT5G63450	58	7	6	6
Cytochrome P450 (CYP704A2**)**^28^	Potri.014G072300	AT2G45510	58	23	19	5
Cytochrome b5 isoform B (CB5-B)^18, 28^	Potri.001G314200	AT2G32720	15	5	5	2
Cytochrome b5 isoform E (CB5-E)^18, 28^	Potri.012G024600	AT5G53560	15	8	7	3
Alcohol dehydrogenase 1 (ADH1)^14, 28^	Potri.002G072100	AT1G77120	41	17	23	15
Alcohol dehydrogenase 2 (ADH2)^14, 28^	Potri.014G193800	AT5G43940	23	5	4	3
Alcohol dehydrogenase 2 (ADH2)^14, 28^	Potri.002G254900	AT5G43940	41	8	7	3
Sterol methyltransferase 2 (SMT2/CVP1)^8, b^	Potri.002G016300	AT1G20330	41	12	12	2
Embryo-specific protein 3 (ATS3)^8, h^	Potri.015G132700	AT5G62200	21	4	4	3
**Tethers/SNARES/Membrane trafficking**						
Synaptotagmin 2 (SYTB; SYT2)	Potri.005G241700	AT1G20080	61	7	5	4
Syntaxin of plants 71 (SYP71) (T-SNARE)^b, h^	Potri.016G088200	AT3G09740	30	5	4	2
Secretion 22 (SEC22) (T-SNARE)^25^	Potri.001G165600	AT1G11890	25	8	3	3
ADP-ribosylation factor A1B (ARF1)^14, 21^	Potri.002G191400	AT5G14670	21	10	9	9
Small GTP-binding protein (ARA-3/RAB8a)^26, b, h^	Potri.008G051700	AT3G46060	24	10	8	7
Golgi localized small GTPase (RAB-6A)^b^	Potri.003G086700	AT2G44610	23	5	6	4
Suppressor of Variegation 11 (SVR11/RABE1B)^b^	Potri.001G110200	AT4G20360	53	9	12	7
Lipid transfer protein (PR-14)PM^TAIR^	Potri.016G135800	AT5G01870	12	3	2	2
Secretion-associated RAS 1/2 (SAR2/SAR1)^22, 30^	Potri.010G141900	AT4G02080	22	20	24	15
COPII vesicle component (Sec24-like)^13^	Potri.005G049100	AT4G32640	111	4	2	2
COPII vesicle component (SAR2)^13^	Potri.010G141900	AT4G02080	22	20	24	15
Coatomer subunit delta (δ-COP/COPI)^13, 18, 28, b^	Potri.012G125500	AT5G05010	58	14	15	4
Coatomer, alpha subunit-1 (COPI)^13, 18, 28, b^	Potri.015G069700	AT1G62020	137	48	36	9
Coatomer subunit beta-1 (COPI)^13, 18, 28, b^	Potri.006G273300	AT4G31480	106	25	19	4
Coatomer subunit gamma (COPI)^13, 18, 28, b^	Potri.004G153500	AT4G34450	99	30	41	6
Guanine Exchange Protein 5 (GEF)	Potri.006G216900	AT3G43300	198	17	17	3
Protein transport protein SEC31B^28^	Potri.009G055400	AT3G63460	123	17	17	4
Rab1 GTPase subfamily (RAB-1B)	Potri.001G080400	AT1G02130	23	13	14	7
RAB GTPase homolog A1F (RAB-A1F)	Potri.001G374000	AT5G60860	24	21	22	9
RAB GTPase homolog A2A (RAB-A2A)^28^	Potri.003G004100	AT1G09630	24	15	14	10
RAB GTPase homolog A2B (RAB-A2B)	Potri.006G000300	AT1G07410	24	10	8	6
Rab-like GTPase (ARA6/RAB5)^20, 23, 25^	Potri.010G226300	AT3G54840	22	7	6	2
RAB GTPase homolog G3D (RAB-G3D/RAB7)^10, 12^	Potri.003G053400	AT1G52280	23	17	13	2
**Organellar**						
Mitochondrial Rho GTPase 1 (MIRO1)^b^	Potri.013G023100	AT5G27540	72	26	24	6
Prohibitin 3 (PHB3)-mitochondrial^25, 28, b^	Potri.001G335700	AT5G40770	31	17	14	13
Prohibitin 6 (PHB6)-mitochondrial^25, h^	Potri.017G017400	AT2G20530	32	16	16	17
V-type proton ATPase subunit a3 (VHA-a3)^b^	Potri.009G121400	AT4G39080	93	11	7	2
V-ATPase C subunit (DET3)^b^	Potri.017G061100	AT1G12840	43	19	21	7
Vacuolar membrane ATPase 10 (AVMA10)^b^	Potri.008G040300	AT3G01390	12	4	4	2
Vacuolar ATP synthase subunit A (VHA-A)^b^	Potri.010G253500	AT1G78900	69	42	38	24
Vacuolar H+-ATPase subunit E1 (VHAE1)^b^	Potri.013G051500	AT4G11150	26	10	8	7
**Heat Shock Proteins/chaperones**						
Endoplasmin homolog (Hsp90-7)^24, b^	Potri.005G241100	AT4G24190	94	33	29	18
DNAJ heat shock family protein (ERDJ3B)^14^	Potri.014G122600	AT3G62600	40	5	3	2
Hsp 70 family protein (BIP2)^b^	Potri.001G087500	AT5G42020	74	37	30	19
Hsp; Endoplasmin homolog (Hsp90-6)^b^	Potri.014G164900	AT3G07770	90	18	18	2
Hsp; Endoplasmin homolog (Hsp90-4)^28^	Potri.006G002800	AT5G56000	80	69	61	28
HSP70-10, Heat shock 70 kDa protein 10^23^	Potri.001G285500	AT5G09590	73	27	25	4
cpn60 (TCP-1), CCT8 [STM, KN1 PD-trafficking]^d, e^	Potri.019G034200	AT3G03960	59	29	24	4
cpn60 chaperonin (TCP-1)^b^	Potri.008G182300	AT1G24510	59	23	12	5
cpn60 chaperonin (TCP-1)^b^	Potri.009G157400	AT3G11830	60	26	20	7
BAG protein 7 (BAG7) (Bcl-2)^28, b^	Potri.015G126800	AT5G62390	46	3	5	3
**Phospolipases-lipases-lipid metabolism**						
Phospholipase C (PLC2)^b^	Potri.010G188800	AT3G08510	67	6	6	2
Phospholipase D delta (PLDδ)^b^	Potri.005G105600	AT4G35790	99	2	2	3
Lipase/GDSL-motif esterase (acyltransferase)^b^	Potri.001G342600	AT5G14450	43	9	10	5
Plat domain protein (PLAT2) (lipase)^8^	Potri.005G076900	AT2G22170	19	4	5	5
Sugar dependent 6 (SDP6)	Potri.010G226700	AT3G10370	69	17	14	2
GDSL-motif esterase (GDSL1) (lipase)^b^	Potri.019G008000	AT1G29670	40	6	4	9
GDSL-like Lipase^b^	Potri.018G089300	AT5G45670	28	10	10	6
Lipoxygenase 2, chloroplastic (LOX2)^c^	Potri.001G015500	AT3G45140	103	6	6	4
Dolichyl-diphosphooligosacch. (HAP6/Rpn2)^28^	Potri.005G226100	AT4G21150	75	10	6	10
Dolichyl-diphosphooligosacch. (HAP6/Rpn2)^28^	Potri.002G036600	AT4G21150	75	12	8	13
Glucose 6-phosph. (GPT1) phosphate transl.1^28^	Potri.011G135900	AT5G54800	43	5	3	2
**Oxidative stress, antioxidant, histones**						
Thioredoxin-dependent peroxidase 1 (TPX1)^h^	Potri.001G423500	AT1G65980	17	9	7	8
Catalase 2 (CAT2)^14, b^	Potri.002G009800	AT4G35090	57	17	14	9
Manganese superoxide dismutase (MSD1)	Potri.013G092600	AT3G10920	25	5	6	4
Peroxidase (PRX36)^b^	Potri.005G195600	AT1G71695	39	15	13	7
Peroxidase (PRX37)^b^	Potri.005G195700	AT1G71695	39	11	9	7
Histone H3 (HTR8, H3.3)^19^	Potri.002G026800	AT5G10980	15	3	2	3
Histone H2A (HTA9, H2A)^19^	Potri.006G249300	AT1G52740	14	3	3	3
Histone H4 (HTA4)^19^	Potri.005G115300	AT5G59970	11	7	5	5

*(1) at LBs or in LB fractions: ^1^Pyc et al. (2017); ^2^Gidda et al. (2016); ^3^Coulon et al. (2020); ^4^Horn et al. (2013); ^5^Kim et al. (2016); ^6^this paper, (Figure 7); ^7^Deruyffelaere et al. (2018); ^8^Kretzschmar et al. (2018); ^9^Rinne et al. (2011);^10^Schroeder et al. (2015); ^11^Ozeki et al. (2005); ^12^Lizaso et al. (2013); ^13^Soni et al. (2009); ^14^Bartz et al. (2007); ^15^Li et al. (2012); ^16^Martin et al. (2005); ^17^Hodges and Wu (2010); ^18^Beller et al. (2008); ^19^Cermelli et al. (2006); ^20^Fujimoto et al. (2004); ^21^Nakamura et al. (2005); ^22^Turro et al. (2006); ^23^Brasaemle et al. (2004); ^24^Umlauf et al. (2004); ^25^Liu et al. (2004); ^26^Sato et al. (2006); ^27^Brocard et al. (2017), ^28^Zhi et al. (2017); ^29^Müller and Ischbeck (2018); ^30^Binns et al. (2006); ^31^Fernández-Santaso et al. (2020), ^32^Wilfling et al. (2013), (2) at PD or in PD enriched fraction: ^a^Liu et al. (2017); ^b^Fernandez-Calvino et al. (2011) (blue); ^c^Karlson et al. (2003); ^d^Xu et al. (2011); ^e^Kitagawa and Jackson (2017); ^f^Knox et al. (2015); ^g^Kriechbaumer et al. (2015); ^h^Leijon et al. (2018) (red)*.

A striking initial finding was that OLEOSIN proteins were not detected in the LB fraction of dormant buds while eight *OLEOSIN* genes were expressed in developing buds (Supplementary Table 1). This was unexpected as we anticipated that OLEOSINs would have remained on the LBs like in desiccated dormant seeds, where they stabilize the monolayer until germination commences (Siloto et al., 2006; Deruyffelaere et al., 2015; Shimada et al., 2018). As OLEOSINs were not detected, they were likely removed from LBs, possibly involving the ubiquitin-mediated 26S degradation system, which removes ubiquitinated OLEOSINs during *Arabidopsis* germination (Deruyffelaere et al., 2015, 2018; Kretzschmar et al., 2018).

**Figure 3:**
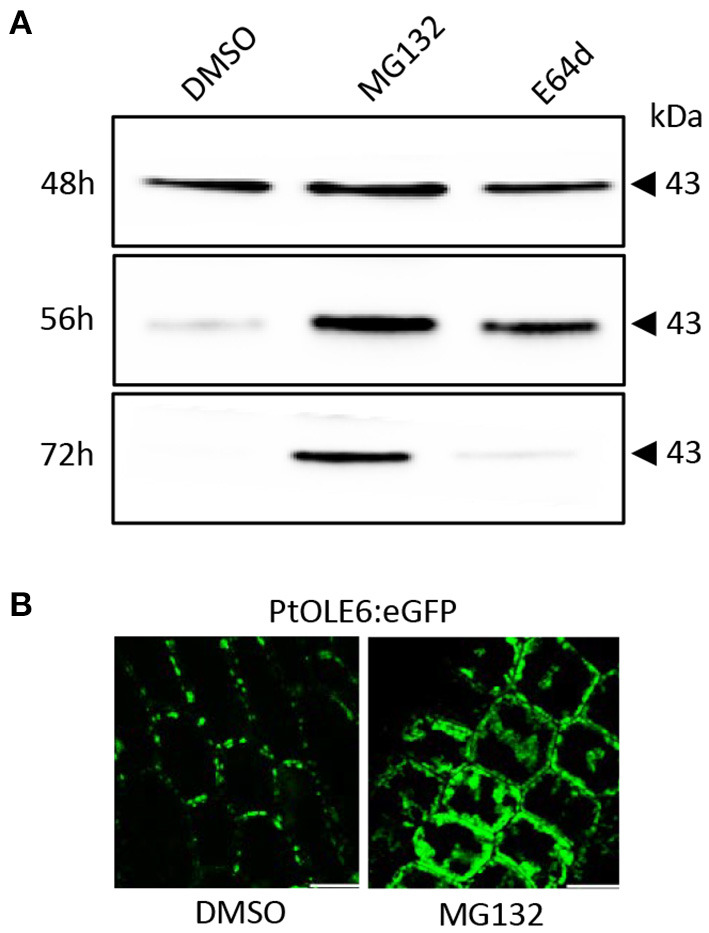
PtOLE6 degradation by the proteasome pathway. **(A)** MG132 inhibits proteasomal degradation of PtOLE6 in germinating *Arabidopsis* seeds. *Arabidopsis* PtOLE6-eGFP overexpressor seeds were germinated in DMSO control, MG132, and E64d for 48–72 h, and immunodetected using an anti-eGFP antibody. **(B)** Treatment with MG132 prevents degradation of PtOLE6-eGFP, resulting in cytosolic accumulation in epidermal cells of the hypocotyl adjacent to the radical. DMSO, control. Bar 50 μm.

To investigate the OLEOSIN disappearance further, we first validated that *Populus* OLEOSINs can be degraded by the ubiquitin-proteasome pathway. For this, we used PtOLE6, as *OLE6* was highly expressed during the LB production phase (Figure 2A). Prediction of PtOLE6 ubiquitination sites with Bayesian Discriminant Algorithm Method (BDM-PUB; http://bdmpub.biocuckoo.org/index.php) showed the presence of at least six putative ubiquitination motifs (Supplementary Figure 5A). In *Arabidopsis*, OLE1-4 displayed one major and one or two minor ubiquitination sites (Deruyffelaere et al., 2015), and their alignment with PtOLE6 shows that the predicted K130 aligns with the major ubiquitination sites of AtOLE3-K159 and AtOLE4-K144 (Supplementary Figure 5B). We next investigated the involvement of the ubiquitin-proteasome-mediated degradation of PtOLE6 by overexpressing *PtOLE6-eGFP* in *Arabidopsis*. While PtOLE6-eGFP was degraded in the controls and the seedlings treated with the vacuolar cysteine protease inhibitor E64d (Deruyffelaere et al., 2015), seedlings treated with the proteasome inhibitor MG132 did not show degradation even after 72 h of imbibition (Figure 3A). Additionally, the MG132 treatment increased cytosolic accumulation of PtOLE6-eGFP (Figure 3B) in cytosolic aggregates, like in the case of AtOLE1 (Deruyffelaere et al., 2015).

Furthermore, we identified key components of the ubiquitin-mediated 26S degradation system in the LB fraction, such as *Populus* homologs of the *Arabidopsis* “plant UBX-domain-containing protein 10” (PUX10) and the segregase Cell Division Control Protein 48A (CDC48A) (Table 1) (Deruyffelaere et al., 2018; Kretzschmar et al., 2018). The bud LB fraction also contained the ubiquitin-activating enzyme UBA1, involved in conjugating ubiquitins to proteins, and several deubiquitinating proteases that prepare the unfolded proteins for degradation by the 20S core protease (Verma et al., 2002) (Table 1, Supplementary Table 1). In addition, the LB fraction contained multiple subunits of the 26S proteasome, such as the crucial 19S ATPase subunit RPT2A, which is important in meristem development (Lee et al., 2011), and 26S scaffolding components (Supplementary Table 1). Although these components are ubiquitous, they are likely to be involved in removing proteins from the LBs, namely, OLEOSINs, as they do so in germinating *Arabidopsis* seeds (Deruyffelaere et al., 2018).

The question remained how LBs retained their structural integrity during their enlargement (Figure 1) while OLEOSINs were absent from the LB fraction (Supplementary Table 1). A possible contributor to stability is the LB protein CALEOSIN1 (CLO1), which was the only CLO identified in the LB proteome (Table 1). However, we identified several other proteins that localize to LBs, such as the recently discovered class II protein Lipid Droplet Interacting Protein (LDIP) (Pyc et al., 2017; Coulon et al., 2020) and the Lipid Droplet Associated Proteins (LDAPs), both of which can contribute to LB stability (Gidda et al., 2016).

### Expression of *PUX10, CDC48A, LDIP*, and *LDAPs*

**Figure 4:**
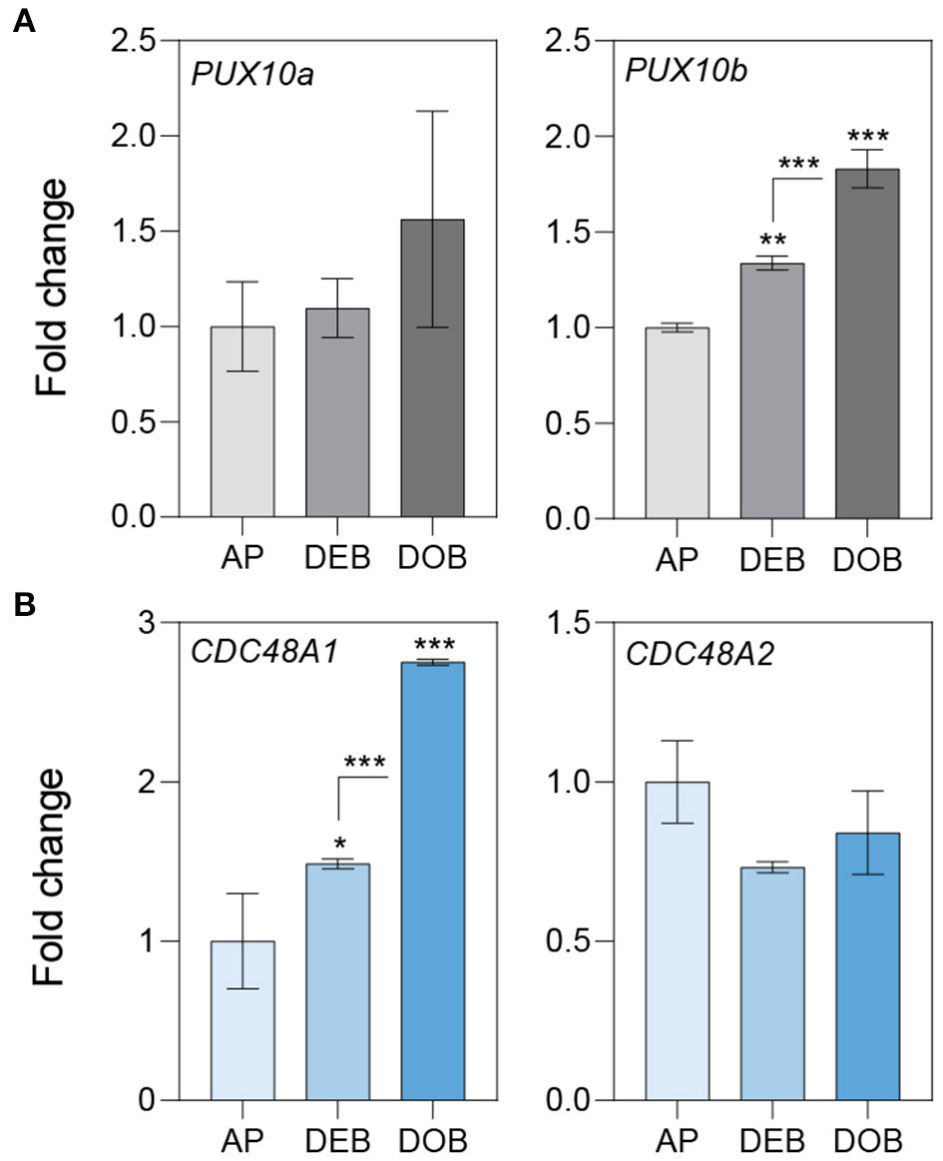
Expression of genes involved in OLEOSIN degradation. Relative expression of genes encoding **(A)** UBX Domain-Containing Proteins (PUX10a, b) and **(B)** Cell Division Control Proteins (CDC48A1,2) in apices (APs) under long day and in developing buds (DEBs) and dormant buds (DOBs) in short days. Values are calculated relative to the control (AP) and represent the means of three technical replicates ± SD of four plants. Asterisk(s) indicate statistical significance between treatments (one-way ANOVA, Tukey analysis; *P < 0.05; **P < 0.01; and ***P < 0.001).

**Figure 5:**
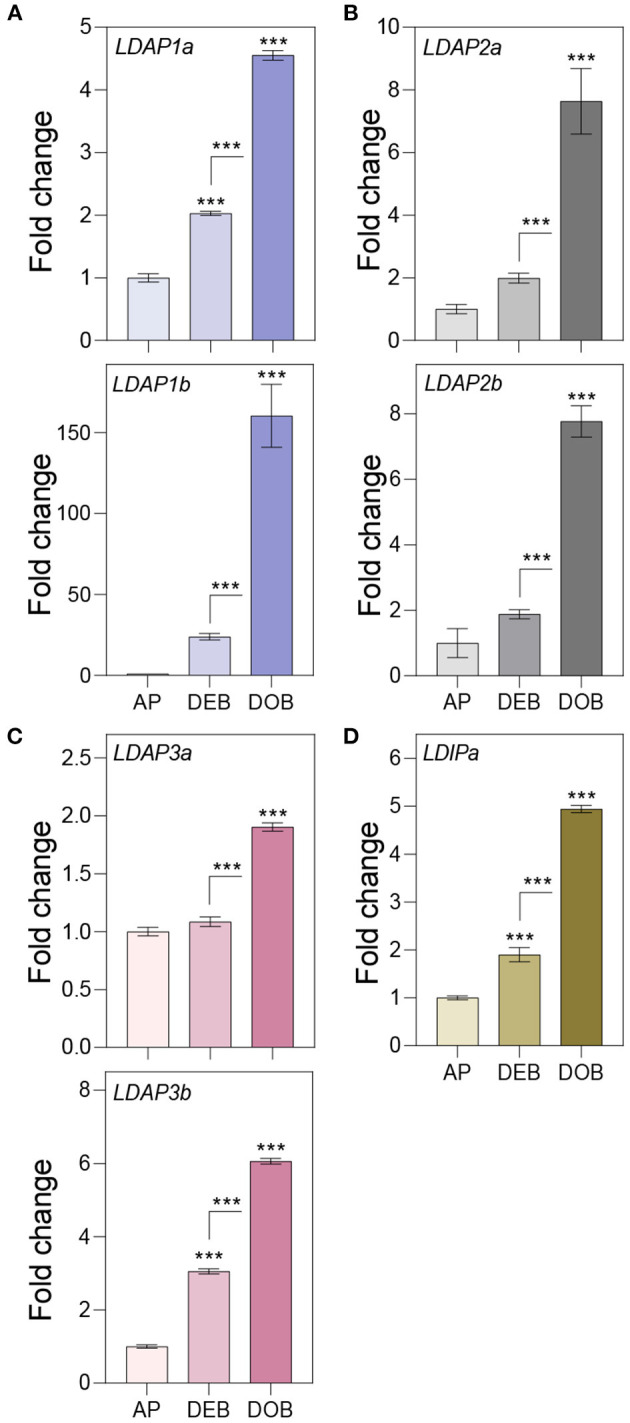
Expression of genes associating with the lipid body monolayer. Relative expression of genes encoding for **(A–C)** LDAPs and **(D)** LDIP in apices (APs) under a long day, and in developing buds (DEBs) and dormant buds (DOBs) in short days. Values are calculated relative to the control (AP) and represent the means of three technical replicates ± SD of four plants. Asterisk(s) indicate statistical significance between treatments (one-way ANOVA, Tukey analysis; ***P < 0.001).

To assess the possibility that OLEOSINs were replaced by LDAPs/LDIP, we identified by phylogenetic analyses two *P. trichocarpa* homologs of PUX10 and CDC48A, six LDAP (LDAP1a, LDAP1b, LDPA2a, LDPA2b, LDAP3a, and LDAP3b), and one LDIP homolog (Supplementary Figures 6–9), and studied their expression in apices during bud development and dormancy establishment. Of the two *PUX10* isoforms, *PUX10b* was upregulated during bud development and further during dormancy development (Figure 4A). Like in the case of *PUX10a*, the expression of one of the *CDC48A* isoforms, *CDC48A2*, was unaltered while *CDC48A1* was upregulated (Figure 4B). The proteins encoded by the upregulated *PUX10b* and *CDC48A1* genes remained present in the LB fraction of dormant buds (Table 1), suggesting they might also remove other ubiquitinated LB proteins. The expression patterns of the six *LDAP* genes were not identical, but all were upregulated during dormancy development, relative to expression levels in apices (Figures 5A–C). Notably, the major *LDAP1b*, which was upregulated during bud development, and excessively high (160-fold) during dormancy development, was little expressed in apices (Figures 5A,C). Also, the genes *LDAP1a* and *LDAP3b* were upregulated during bud development, while *LDAP3a* was not (Figure 5C). *LDAP2a* and *LDAP2b* were only slightly upregulated during bud development (Figure 5B). At this point, *OLE6* was still very highly expressed (Figure 2A). The timeline shows that during dormancy development, when *OLE*6 was downregulated below the expression level in apices, all the five *LDAP* genes were strongly upregulated, especially *LDAP1b* (Figure 5A). In contrast, the upregulation of *LDIP* (ca. 5-fold; Figure 5D) was comparable to that of the other *LDAP*s.

### Presence of LDAP1 in Maturing Buds and Localization at LBs

**Figure 6:**
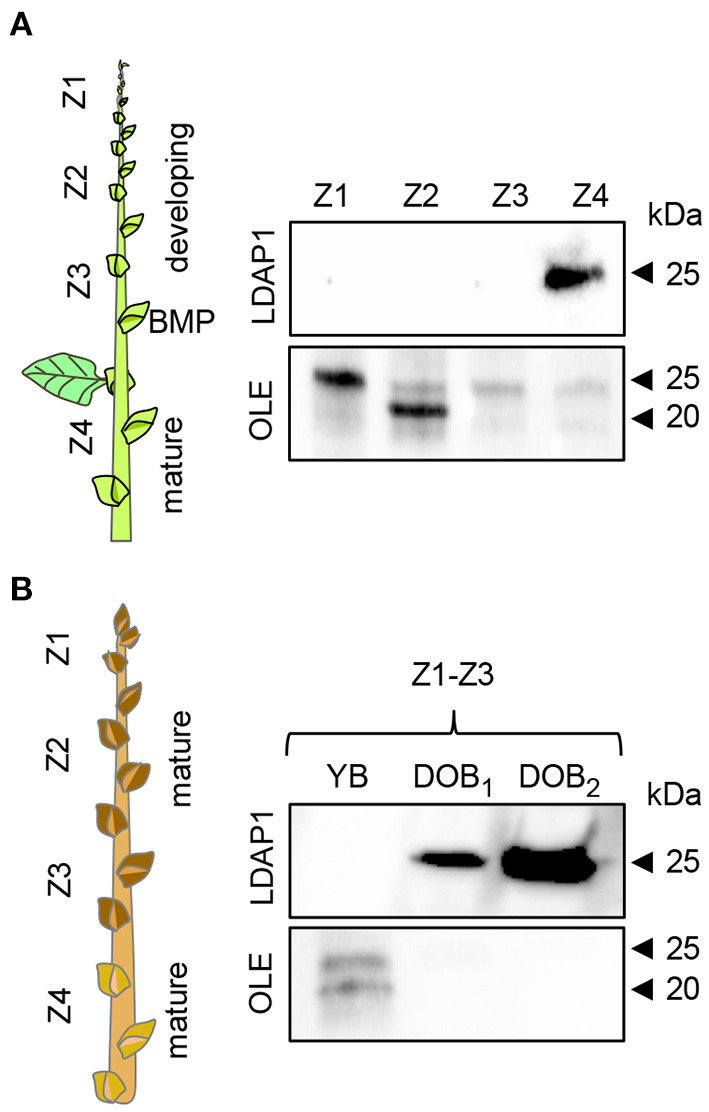
Immunodetection of LDAP1 and OLEOSIN proteins in axillary buds during their maturation under different daylength conditions. **(A)** Developing (zones Z1–Z3) and mature axillary buds (Z4) in reference to the bud maturation point (BMP) in long days. **(B)** Young axillary buds (YB) (Z1–Z3) in long days, and after 6 (DOB_1_, dormant buds 1) and 9 weeks (DOB_2_, dormant buds 2) in short days. Equal amounts of protein (10 mg) were loaded in wells.

In addition to the gene expression studies, we investigated if OLEOSIN and LDAP were present in extracts of buds at different developmental stages during growth and under dormancy-inducing conditions. In growing plants, antibodies detected OLEOSINs in younger developing buds above the bud maturation point (BMP), while LDAP1 was not detected (Figure 6A). In contrast, LDAP1 appeared to be abundant in full-grown buds below the BMP, which had ceased development and entered quiescence. Under dormancy-inducing conditions, OLEOSINs could not be detected in any bud, whereas LDAP1 was found in dormant buds and was even increasing over time in dormant buds (Figure 6B).

**Figure 7:**
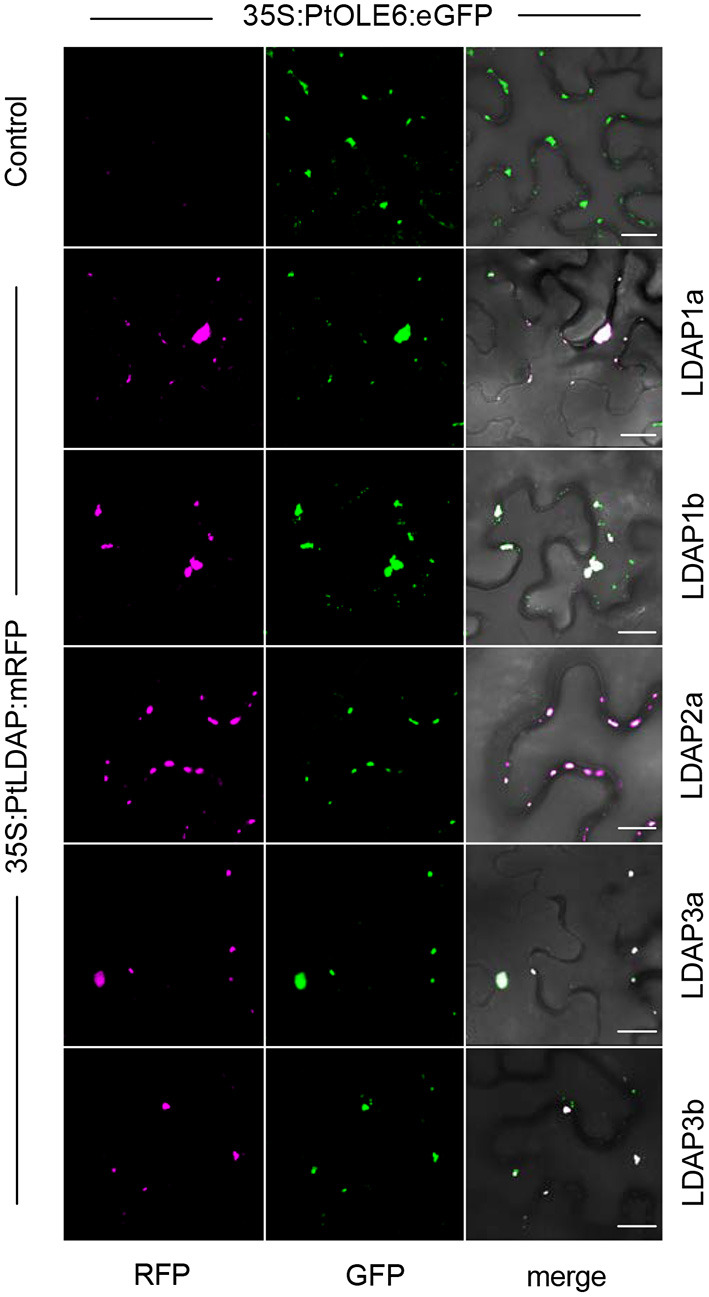
Localization of Lipid Droplet Associated Proteins (LDAPs) at lipid bodies in *N. benthamiana*. Representative confocal laser scanning microscopy images of leaf epidermal cells transiently co-expressing eGFP-tagged OLE6 and corresponding mRFP-tagged LDAPs (magenta). Single optical sections of RFP, GFP, and the merged images are shown for each set of experiments. Bars 50 μm.

That the *Populus* LDAPs can localize to LBs was demonstrated in leaf epidermal cells of *N. benthamiana* using a binary vector expressing the 35S promotor driven *OLE6-eGFP* fusion protein and binary vectors expressing *LDAP1a-mRFP, LDAP1b-mRFP, LDAP2a-mRFP, LDAP3a-RFP*, and *LDAP3b-mRFP*. The vectors were transformed into *Agrobacterium tumefaciens* and infiltrated into leaves for 2 days and investigated with CLSM. Captured images (Leica Application Suite X software) showed that all mRFP-tagged LDAPs were exclusively localized to OLE6:eGFP-tagged LBs (Figure 7). LDAP1b and LDAP3b did not localize to the smallest LBs, which only contained OLEOSIN. In brief, the upregulation of *LDAPs*, LDAPs presence in the LB fraction, and localization to LBs suggest that they were recruited to the monolayer after OLEOSIN removal. Together, these results suggest that OLEOSINs were degraded and replaced by LDAPs prior to bud completion and the establishment of a quiescent and dormant state.

### Candidate LB-Associated Proteins

The proteomic analysis of the bud LB fraction aimed to create an inventory of known and putative LB cargos that may contribute to cellular homeostasis, dormancy release and subsequent intercellular transport, and signaling in meristems. Their detection requires the omission of the salt wash (as shown above) that is commonly used to remove proteins that might associate with the monolayer during LB isolation, or are commonly considered contaminants. As argued above, this would also remove the peripherally associated proteins that hitch a ride to the PD. In buds, cytoplasmic proteins might associate with LBs when molecular crowding is reduced (Kory et al., 2015), and it seems possible that the enlarging LBs recruited a surplus of such proteins. These proteins may be stored at LBs and delivered to the PM and PD during dormancy release, serving membrane repair and renewed cell–cell communication. From the total fraction of 719 proteins, we selected 117 proteins, or ca. 16%, that potentially represent meaningful LB cargos (Table 1). As indicated, this selection was based on the reported presence of identical or similar proteins at LBs, in LB fractions, at PD, or in PD-enriched fractions (Fernandez-Calvino et al., 2011; Leijon et al., 2018). Since LBs frequently interact with other cell organelles and PD, we also included proteins that were reported or suggested to assist in LB-organelle-PM tethering, targeting, and hemifusion.

We detected in the LB fraction Caleosin 1 (CLO1), LDPI, LDAP3a, LDAP3b, LDAP1a, LDAP1b, Oil Body Lipase1 (OBL1a,b), GPAT8 (functionally redundant with GPAT4), and the 1,3-β-glucanase enzyme GH17_44 (Table 1). Like OLEOSIN, CLO1 has three structural domains, such as a pro-knot motif (Hsieh and Huang, 2004). Its major function is signaling, but at LBs, it contributes to monolayer stability (Chen et al., 1999). LDAPs associate with LB *via* amphipathic helices and similarly provide some structural integrity (Horn et al., 2013; Gidda et al., 2016; Kim et al., 2016). LDIP, which possesses a central hydrophobic sequence and few TMDs (Pyc et al., 2017), interacts with LDAP3 (Pyc et al., 2017) and may associate with nascent LBs (Coulon et al., 2020). OBL1 localizes to LBs in *Arabidopsis* seeds (Eastmond, 2006), and GH17_44 localizes to LBs in *Populus* meristems (Rinne et al., 2011). PUX10 and CDC48A are involved in the degradation of ubiquitinated proteins, such as OLEOSINs and might end up in the LB fraction attached to LB proteins. The LB fraction contained a dozen components of the 26S proteasome (Verma et al., 2002), such as the regulatory particle RPT2A and regulatory subunit RPN7, the ubiquitin-conjugating enzyme 13A (UBC13A), the ubiquitin-activating enzyme E11 (UAB1) (Table 1), and other 26S-associated proteins (Supplementary Table 1).

As LBs move in the actomyosin system motorized by myosin XI-k/1/2 (Veerabagu et al., 2020), it is of interest that the LB fraction contained two myosin XI tail binding GTPases. RAB-C2a, a homolog of the mammalian RAB18 that localizes to LBs, localizes in plants to peroxisomes that traffic on F-actin, like LBs (Hashimoto et al., 2008). RAB-D1 localizes to Golgi and endosomal vesicles (Pinheiro et al., 2009), which can interact with LBs (see discussion), while ARA6 localizes to LBs (Brasaemle et al., 2004; Fujimoto et al., 2004; Liu et al., 2004). The LB fraction contained only one actin, ACT7. This could be significant as ACT7 is associated with meristematic activity in germination and early plant development (McDowell et al., 1996; Kandasamy et al., 2001, 2009), is required in callus growth and present in PD fractions (Fernandez-Calvino et al., 2011). We also identified proteins that modulate actin dynamics, such as ADF4 and VAB2 (Table 1).

Proteins in the LB fraction that localize to PD components or the surrounding PM include, among others, (references in Table 1) Purple Acid Phosphatase (PAP1), two calreticulins (CRT1/CRT1A and CRT2/CRTB), two peroxidases (PRX36 and PRX37), the atypical 1,3-β-glucanase Zerzaust (ZET), 1,3-β-glucanase1 (BG1/GH17-37), Dehydrin HIRD11, Reticulon like protein B1 and B3 (RTN3), an inactive receptor kinase, Plasma Membrane Intrinsic Protein 1 and 3 (PIP1 and 3), Calnexin 1 (CNX1), and Bcl-2 Unfolding Protein BAG7 (Table 1; Supplementary Table 1). Of these, five were present in the *Populus* PD fraction (Leijon et al., 2018) and nine in that of *Arabidopsis* (Fernandez-Calvino et al., 2011). Several of these proteins were also previously localized to LBs, such as CNX1, RTN1, and RTN3 (Table 1).

Proteins previously found in LB fractions of plant and non-plant systems included LEA proteins, which protect cellular structure and PD during dehydration stress (Karlson et al., 2003). Among these were ERD7, LEA26, and LEA27 (Table 1). Furthermore, we identified alcohol dehydrogenases (ADH1-3), annexins (ANN1,2), transport family protein SecY, sterol methyltransferase (SMT2), embryo-specific protein ATS3, and Cytochrome P450 (Table 1).

The LB fraction also contained proteins involved in tethering, membrane trafficking, and membrane fusion, such as the CalB domain Synaptotagmin 2 (SYTB/SYT2), which is a C2 tethering protein, Syntaxins (SYP71) and other SYPs, the vesicle/protein transporter ADP-ribosylation factor 1 (ARF1), and the endosomal protein Guanine Nucleotide-Exchange (GEF), which recruits ARF1 to vesicles (Table 1, Supplementary Table 1). Moreover, a dozen Rab GTPases were present. These included ARA-3 (RAB8a), Golgi localized RAB-6A, and Suppressor of Variegation 11 (SVR11/RABE1B) (Table 1, Supplementary Table 1). The presence of CPOII subunits and COPI Coatomer subunits in conjunction with ARF1 is of interest, as they may mediate protein trafficking to and from LBs (Soni et al., 2009) (see discussion).

Other identified proteins potentially reflect organellar interaction, protein folding and unfolding at organelles and PD, lipid metabolism, storage, detoxification, and desiccation stress. Unfolding/folding proteins in the bud LB fraction included the chaperones/Heat Shock proteins Hsp-90-4, Hsp90-6, Hsp90-7, the Hsp70 proteins BIP2 and BIP3, and homologs of the cpn60 chaperonin TCP-1/CCT8. Of the TCP-1 chaperonin family proteins, 10 members were present (Table 1, Supplementary Table 1). Proteins related to lipid metabolism included Phospholipase C (PLC2), GDSL-motif esterase (acyltransferase), and the lipase Plat Domain Protein 2 (PLAT2). Also identified were three Histones, previously shown to be stored at LBs (Cermelli et al., 2006). The identified antioxidant enzymes may protect stem cells of the embryonic shoot from hypoxia-induced damage in the low-oxygen environment of the bud (Ophir et al., 2009; Meitha et al., 2015). They include Catalase 2 (CAT2), Thioredoxin-dependent peroxidase 1 (TPX1), the peroxidases PRX36 and PRX37, and Manganese Superoxide Dismutase (MSD1).

A direct comparison of the LB fraction of dormant buds and the PD fraction of cell suspension cultures of *P. trichocarpa* (Leijon et al., 2018) uncovered 19 identical proteins (Table 2). A part of these shared proteins was also identified in the PD fraction of *Arabidopsis* cell suspensions (Table 2). The presence of LDIP in the *Populus* PD fraction (Leijon et al., 2018) is of interest because LDIP is a LB protein (Pyc et al., 2017). Of interest is also the syntaxin SYP71, a plant-specific SNARE protein involved in vesicle docking and fusion (Suwastika et al., 2008) that is present in PD fractions of both *Populus* and *Arabidopsis* (Table 2). SNAREs can mediate LB tethering to distinct compartments, LB-LB fusion, and hemifusion with bilayer membranes (Boström et al., 2007; Murphy et al., 2009). The GTPase ARA-3 (RAB8a), which mediates vesicle docking, the PD-localized Calreticulin (CRT1/CRT1A) (Baluska et al., 2001), and the LEA protein ERD4 were present in both fractions. Likewise, the following proteins of the bud LB fraction were present in both PD fractions: ZET (Vaddepalli et al., 2017), an O-Glycosyl hydrolases family 17 protein, and the GPI-anchored protein SKU5 similar 1 (SKS1). Some proteins exclusively shared by the LB fraction and the *Populus* PD fraction were the Thioesterase superfamily protein, involved in fatty acid biosynthesis, the seed LB candidate protein Embryo-Specific Protein 3 (ATS3) (Vermachova et al., 2011; Kretzschmar et al., 2020), a leucine-rich receptor kinase (probably inactive), PAP1, the cytoplasmically localized Reversible Glycosylated Polypeptide 3 (RGP3), the aquaporin Plasma Membrane Intrinsic Protein 1 (PIP1), the PHB-domain protein PHB6, the antioxidant TPX1, and the hydrolase S-Formylglutathione Hydrolase (SFGH).

**Table 2:** Proteins that are shared by the bud LB fraction of *Populus* and the PD-enriched fraction of *Populus trichocarpa* Leijon et al. (2018).

**Protein name**	***P. trichocarpa* Accession nr**	**BlastP of *A. thaliana***	**Mw**	**Unique Peptides**		
			**kDa**	**Bio 1**	**Bio 2**	**Bio 3**
LDAP Interacting Protein (LDIP)	Potri.004G082300	AT5G16550	24	3	3	4
CSC1-like protein ERD4 (ERD4) (LEA)	Potri.001G358300	AT1G30360*	82	9	5	5
Syntaxin of plants 71 (SYP71)	Potri.016G088200	AT3G09740*	30	5	4	2
Small GTP-binding protein (ARA-3) (NAC)	Potri.008G051700	AT3G46060*	24	10	8	7
Leucine-rich repeat protein kinase	Potri.018G074300	AT2G26730	71	9	3	2
Calreticulin 1A (CRT1/CRT1A)	Potri.005G015100	AT1G56340*	48	13	10	4
Purple acid phosphatase (PAP1)	Potri.010G158400	AT1G13750*	69	3	3	2
Reversibly glycosylated polypeptide 3 (RGP3)	Potri.010G156700	AT3G08900	41	36	32	11
Zerzaust (ZET), atypical β-1,3 glucanase	Potri.019G032900	AT1G64760*	53	4	4	2
O-Glycosyl hydrolases family 17 protein	Potri.006G080600	AT5G58090*	53	5	6	2
Embryo-specific protein 3 (ATS3)	Potri.015G132700	AT5G62200	21	4	4	3
Plasma membrane intrinsic protein 1 (PIP1)	Potri.003G128600	AT4G00430	31	6	4	2
SKU5 similar 1 (SKS1)	Potri.015G127200	AT4G25240*	66	10	6	4
Thioredoxin-dependent peroxidase 1 (TPX1)	Potri.001G423500	AT1G65980	17	9	7	8
S-formylglutathione hydrolase (SFGH)	Potri.006G047000	AT2G41530	32	6	7	2
Prohibitin 6 (PHB6)	Potri.017G017400	AT2G20530	32	16	16	17
Thioesterase superfamily protein	Potri.003G020300	AT5G10160*	26	5	4	4
Ribosomal protein L10 family protein	Potri.008G066200	AT2G40010*	34	8	9	6
Ribosomal protein L7Ae/L30e/S12e (Gadd45)	Potri.004G196500	AT1G36240	12	4	2	2

Proteins with a star* are also present in the PD-enriched fraction of Arabidopsis thaliana (Fernandez-Calvino et al., 2011).

## Discussion

### Production and Degradation of OLEOSINs

OLEOSINs stabilize the LB monolayer, preventing coalescence and fusion during seed desiccation and dormancy (Siloto et al., 2006; Shimada et al., 2008; Hsiao and Tzen, 2011). Perennial buds also desiccate, albeit partially (Rinne et al., 2015), express *OLEOSIN* genes, produce OLEOSINs, accumulate LBs, and establish dormancy. The major gene *OLE6* was strongly upregulated during bud formation (Figure 2A). Given these similarities, it came as a surprise that none of the eight *Populus* OLEOSINs were detected in the LB fraction of dormant buds (Table 1; Supplementary Table 1). *OLE6* was completely downregulated prior to dormancy establishment (Figure 2A), while the minor genes *OLE3* and *OLE5* were expressed very little. The apparent absence of OLEOSINs in the LB fraction of dormant buds suggests they were degraded prior to dormancy establishment, likely by the machinery that also degrades OLEOSINs during seed germination (Deruyffelaere et al., 2018). Core components of this machinery were found in the *Populus* LB fraction, such as the adaptor protein PUX10, the segregase CDC48A (AAA ATPase Cell Division Cycle 48) and various 26S components, the ubiquitin-activating enzyme UBA1, the crucial 19S ATPase subunit RPT2A, and several deubiquitinating proteases, which are required for insertion of proteins into the 20S core (Table 1, Supplementary Table 1). PUX10 associates with LBs, and interacts with ubiquitin through its UBA domain and with CDC48A through its UBX domain, resulting in the degradation of ubiquitinated OLEOSINs by the 26S proteasome (Hsiao and Tzen, 2011; Deruyffelaere et al., 2015, 2018; Kretzschmar et al., 2018). As AtPUX10 is an integral class I LB protein that binds ubiquitins and recruits CDC48A (Deruyffelaere et al., 2018), it is perhaps not surprising that these components were present in the LB fraction of *Populus* buds. The predicted K130 ubiquitination site of PtOLE6 aligned with the major ubiquitination sites of AtOLE3-K159 and AtOLE4-K144 (Supplementary Figure 5B). Moreover, we found that in germinating seeds of an *Arabidopsis, PtOLE6* overexpressor PtOLE6 is degraded like AtOLE1-4 (Deruyffelaere et al., 2015), and that degradation is partially inhibited by proteasome inhibitors E64d and blocked by MG132 (Figure 3). It seems likely, therefore, that this degradation mechanism is responsible for the early removal of OLEOSINs from LBs of buds, although direct localization of these proteins to the LBs remains to be demonstrated. During bud formation, the major gene *OLE6* was strongly upregulated, producing OLEOSIN protein and many small LBs (Figures 1, 2). During dormancy establishment, *OLE6* was downregulated below the level of growing apices (Figure 2A); and while *PUX10b* and *CDC48A1*were upregulated (Figures 4A,B), OLEOSINs were no longer detected (Figure 6B), and LBs had enlarged (Figure 1E). In correspondence with *OLE6* downregulation, *LDIPa, LDAP1, LDAP2*, and *LDAP3* were upregulated (Figure 5); and their encoded proteins, LDIP, LDAP1, and LDAP3, were detected in the bud LB fraction instead of OLEOSINs (Table 1). In brief, the data support the hypothesis that OLEOSIN is required for initiation of LB production in buds, but that prior to dormancy establishment OLEOSINs are removed by the PUX10/CDC48a degradation mechanism. PUX10 and CDC48A also likely degrade other LB proteins (Deruyffelaere et al., 2018), as they remain in LBs after OLEOSIN is removed.

### Probable Replacement of OLEOSIN by LDIP/LDAPs

That OLEOSINs are replaced by LDAP1 during LB enlargement is supported by the following facts. First, in long-day plants, OLEOSIN was detectable in extracts of developing buds and virtually absent in mature buds, while the reverse was true for LDAP1 (Figure 6A). Second, in short-day plants, OLEOSINs could not be detected in dormant buds, while LDAP1 was abundant (Figure 6B). Indeed, in general LDAPs might not interact with OLEOSIN-covered LBs, because LDAPs do not possess a hydrophobic hairpin and their relative hydrophilicity allows LB association only in detergent-sensitive manner (Gidda et al., 2016; Kim et al., 2016; Huang and Huang, 2017; Pyc et al., 2017). LDAPs and many of the identified LB fraction proteins might have been recruited to the expanding monolayer surface after OLEOSIN degradation, such as CLO1 (Table 1), which binds competitively with the monolayer (Huang, 2018). Noteworthy is that in *N. benthamiana*, the mRFP-tagged LDAP1b and LDAP3b, did not localize to the smallest OLE6-eGFP-tagged LBs (Figure 7), suggesting that these two proteins associate only with an expanded monolayer. LDIP anchors itself *via* an amphipathic helix in the monolayer and interacts with LDAP3 (Pyc et al., 2017) but is confined to a small area of the LB while LDAPs cover the entire monolayer (Coulon et al., 2020). LDIP was suggested to facilitate the neo-formation of small LBs (Coulon et al., 2020) in cooperation with SEIPINs (Cai et al., 2015; Barbosa and Siniossoglou, 2017). As *LDIPa* was upregulated during the LB accumulation phase in developing buds (Figure 5D), it seems possible that it did assist later LB emergence. LB expansion might be driven by increased TAG biosynthesis (Figures 1E, 2B), possibly involving the ER-localized enzyme GPAT8 (Table 1) (Gidda et al., 2009), which functions redundantly with GPAT4 (TAIR). Both localize to LBs in *Arabidopsis* (Fernández-Santaso et al., 2020) and in mammalian and insect cells (Wilfling et al., 2013).

Seed germination involves OLEOSIN degradation, LB enlargement, and recruitment of cytoplasmic proteins that mediate lipolysis and promote seedling growth (Deruyffelaere et al., 2015; Thazar-Poulot et al., 2015; D'Andrea, 2016). Germination cannot occur when the TAG lipase SDP1 is absent (Eastmond, 2006). While in buds *SDP1a* and *SDP1b* were slightly upregulated under short days (Figure 2C), the enzymes were absent from the LB fraction (Supplementary Table 1), and LB degradation was not observed. Either way, *SPD1* transcript levels might not reflect enzyme activity because it might be post-transcriptionally regulated (Eastmond, 2006), or it is not delivered to LBs by the glyoxysomes that produce them (Thazar-Poulot et al., 2015) but kept in store for later bud activation. Notably, the retromer subunit VPS29 that mediates peroxisome tubulation to deliver SPD1 to the LBs was absent from the LB fraction (Supplementary Table 1).

### LB Fraction and Putative LB Proteome

LBs function in lipid homeostasis, protein sequestration, and membrane and cargo trafficking (Murphy, 2012). As a result, LB fractions can contain tens or even hundreds of proteins (Bartz et al., 2007; Hodges and Wu, 2010; Brocard et al., 2017; Zhi et al., 2017), most of which lack canonical LB functions. These “refugee” proteins (Hodges and Wu, 2010) are of interest, highlighting the dynamics of protein exchanges. Our proteomic analyzes were aimed at creating an inventory of such transiently associated proteins, as they could be involved in cellular homeostasis, dormancy release, and intercellular transport and signaling. The 719 identified proteins were present in the LB fractions of three independent experiments (Table 1, Supplementary Table 1), and among these were 117 proteins that included known LB proteins and proteins that were previously identified in LB fractions of various systems (Table 1). They relate to the ER, lipid metabolism, cytoskeleton, membrane trafficking, organellar interaction, membrane tethering and fusion, chaperone function, and signaling (Zehmer et al., 2009; Hodges and Wu, 2010; Gao and Goodman, 2015). Some of them might serve the unique needs of dormant bud meristems, which have reduced metabolism, face environmental and endogenous stresses, and maintain a dormancy-release mechanism (Rinne et al., 2001, 2011). In the absence of OLEOSINs, which mitigate desiccation and freezing stress (Siloto et al., 2006; Shimada et al., 2018), LDAPs, CLO1, and hydrophilins, such as LEA proteins (Table 1), might confer stability to LBs (Gidda et al., 2016; Kim et al., 2016). The bud, as a survival structure, must protect the enclosed embryonic shoot and its resident stem cells against environmental and endogenous onslaughts. Considering that the enclosed meristem resides in a hypoxic internal bud environment (Ophir et al., 2009; Meitha et al., 2015), the identified antioxidants might function to protect the stem cells. Notably, the LB fraction contains TPX1, the peroxidases PRX36, PRX37, CAT2, and MSD1 (Table 1), which, like in *Drosophila* (Bailey et al., 2015), can mitigate the effects of hypoxia-induced ROS. LBs may also detoxify stem cells by removing cytoplasmic free fatty acids, resulting from membrane degradation and remodeling during desiccation (Listenberger et al., 2003). Given the embryo-like nature of the embryonic shoot, the presence of histones H2A, H3, and H4 in the LB fraction is significant. *Drosophila* stores maternal H2A, H2B, H3, and H4 at LBs in its eggs as a repository for early embryogenesis (Cermelli et al., 2006; Li et al., 2012). Storage at LBs keeps histones available for immediate use while avoiding the cytotoxic effects of free histones (Gunjan and Verreault, 2003). In bud meristems, they could serve a similar purpose.

### LB Fraction and LB Trafficking to PD

As shown previously, LBs do not arrive at PD by simple cytoplasmic streaming but by processive trafficking in the actomyosin system, which is severely impaired in an *Arabidopsis* 3KO *myosinxi-k/1/2* mutant (Veerabagu et al., 2020). Myosin XI-K is the most important among the 13 class XI myosins of *Arabidopsis* and is responsible for the movement of mitochondria, peroxisomes, and endomembrane vesicles (Avisar et al., 2008, 2009; Sparkes et al., 2008; Peremyslov et al., 2015). The binding of LBs to myosin XIs, like that of other organelles, might require Rab GTPases. We identified in the LB fraction two “myosin XI tail-binding” proteins, the Rab GTPase homolog C2A (RAB-C2A) and the Rab GTPase homolog D1 (RAB-D1) (Table 1). In *Arabidopsis*, these factors interact with the C-terminal tail region of MYA2 (an isoform of myosin XI). While RAB-C2A specifically localizes to peroxisomes (Hashimoto et al., 2008), RAB-D1 associates with the Golgi apparatus (Pinheiro et al., 2009). A recent study on the *Populus* Rab family found that RAB-C2A did not localize at Golgi, TGN, or endosomes but instead at small unidentified vesicles (Zhang et al., 2018), which could have been peroxisomes but possibly also LBs. The presence of RAB-C2A in the LB fraction (Table 1) suggests that RAB-C2A is the receptor that anchors LBs to myosin XI-k/1/2, enabling motility, interaction with cell organelles, and targeting of PD. If so, the motility of LBs and peroxisomes on the same RAB-C2A/Myosin XI/actin assembly might explain their frequently observed interaction (Mathur et al., 2002; Hashimoto et al., 2008; Veerabagu et al., 2020). Notably, RAB18, a mammalian homolog of RAB-C2A, is present at LBs and in LB fractions (as shown in references in Table 1). The presence of Golgi-related RAB-D1 in the LB fraction (Table 1) suggests an interaction between LBs and Golgi- and endosomal vesicles (see below). LBs might move on ACT7, as it is the only actin identified in the bud LB (Table 1) and seed LB fractions of Chinese tallow (Zhi et al., 2017). ACT7 is required in seed germination, early plant development, and meristem proliferation (McDowell et al., 1996; Kandasamy et al., 2001, 2009), and it is found in the PD fraction of *Arabidopsis callus* cells (Fernandez-Calvino et al., 2011). The presence of actin-stabilizing VAB2 (Ma et al., 2012) and PD-localized actin-severing ADF4 in the LB fraction (Table 1) appears to reflect the dynamics of actin-mediated transport.

### Organellar Interactions and Chaperones

As argued by Gao and Goodman (2015), organelle markers, such as ER luminal chaperones and mitochondrial proteins, are so frequently associated with thoroughly purified LBs that it is unlikely that they all represent contaminants. At least some of them might reflect the frequently observed LB-organellar interactions (Murphy et al., 2009; Zehmer et al., 2009), facilitated by their movement on F-actin (Mathur et al., 2002; Van Gestel et al., 2003; Hashimoto et al., 2008; Veerabagu et al., 2020). We identified some mitochondrial proteins, vacuolar proteins, and lipid metabolism proteins, as well as Annexins, Calnexin, LEA proteins, Cytochrome P450s, transport proteins, Alcohol Dehydrogenases, and Embryo-Specific Protein3 (Table 1, Supplementary Table 1). Several of these are present in seed LB fractions and PD fractions (as shown in references in Table 1). The identified annexins putatively link LBs to the PM and PD, as they can bind F-actin, regulate membrane trafficking, bind phospholipids and Ca^2+^, inhibit callose synthase at PD, act as peroxidases, and induce membrane curvature (Delmer and Potikha, 1997; Clark et al., 2012; Boye et al., 2018).

Organellar interactions involve chaperone-like molecules that mediate membrane and vesicle transport, tethering, and membrane (hemi-)fusion. Chaperones transport, fold and unfold proteins during autophagy, and function at peroxisomes, mitochondria, chloroplasts, and ER (Boston et al., 1996; Kriechbaumer et al., 2012). We identified Synaptotagmin B, various Syntaxins, ARF1 and ARF-related proteins, and transport-related RAB GTPases (Table 1). The identified calnexins and several of the chaperones (Table 1) were also present in the LB fraction of the Chinese tallow (Zhi et al., 2017). In the SAM, LB-resident chaperones, delivered to PD, could assist in the documented unfolding and refolding of the non-cell-autonomous proteins that move cell-to-cell (Aoki et al., 2002). Essential in SAM function is the intercellular movement of the conserved transcription factor SHOOT MERISTEMLESS (STM) in *Arabidopsis*, and KNOTTED1 (KN1) in maize, which requires chaperon-assisted unfolding in the donor cell and chaperonin-assisted refolding in the destination cell (Kitagawa and Jackson, 2017). The LB fraction contained, among others, the CCT8 subunit of chaperonin CTP-1 (AT3G03960) that refolds STM/KN1 in the destination cell (Xu et al., 2011; Kitagawa and Jackson, 2017). Although CCT8/CTP-1 localization at LBs remains to be shown, LBs that dock at opposite sides of PD (Rinne et al., 2011; Veerabagu et al., 2020) could engage in unfolding proteins for export and refolding incoming proteins in the SAM.

### LB Proteins Localized to PD or Present in PD-Enriched Cell Wall Fractions

Lipid bodies can target PD to deliver enzymes that enhance PD conductivity (Rinne et al., 2001, 2011; Veerabagu et al., 2020). Part of the identified proteins may similarly arrive at the PD interior and surrounding PM *via* the LB shuttle. This seems feasible, as many of the proteins in the LB fraction have been localized to PD, such as acid phosphatases (Esau and Charvat, 1975), calreticulins (Baluska et al., 2001), calnexins (Liu et al., 2017), and reticulon (Knox et al., 2015; Kriechbaumer et al., 2015).

Reticulon, present in the LB fraction of insect cells, adopts a hairpin-like topology in the outer ER leaflet (Beller et al., 2008). It is of interest, therefore, that reticulon3 (RTN3) is present in the bud LB fraction (Table 1), and that it is identified in the PD fraction (Fernandez-Calvino et al., 2011) and localized at PD (Knox et al., 2015; Kriechbaumer et al., 2015). This suggests the possibility that in meristems RTN3 could arrive at the desmotubule *via* LBs. The dehydrin HIRD11 (Table 1) may protect PM- and PD-localized proteins during winter, such as the PD-localized dehydrin in cold-acclimated tissue of dogwood (Karlson et al., 2003). Similarly, the γ-clade GH17-family protein (Table 1) localizes to PD (Rinne et al., 2011). RGP3 might also localize to PD, like RGP2 (Sagi et al., 2005). Proteins in the LB fraction, some of which localized to PD, were also identified in PD fractions of both *Arabidopsis* (Fernandez-Calvino et al., 2011) and *P. trichocarpa* (Leijon et al., 2018), such as ERD4, Syntaxin of Plants 71 (SYP71), small GTP-binding protein ARA-3 (NAC/RAB8a), Calreticulin 1A (CRT1A), PAP1, ZET, O-Glycosyl hydrolases family 17 protein, SKU5 similar 1 (SKS1), and Thioesterase superfamily protein (Table 2). The presence of the LB protein LDIP and the candidate LB protein ATS3 (Vermachova et al., 2011; Kretzschmar et al., 2020) in the *Populus* PD proteome (Leijon et al., 2018) and in the LB fraction (Table 1) is an independent confirmation that LBs target PD.

The number of proteins in the LB fraction that are destined for PD might be much larger than that which is found in enriched PD fractions of cell cultures because they contain only young primary PD (Fernandez-Calvino et al., 2011; Leijon et al., 2018). In complex three-dimensional tissues, such as bud meristems, LBs dock at both primary and secondary PD (Rinne et al., 2011; Veerabagu et al., 2020). The overlap with available PD proteomes might, therefore, underestimate the LB proteins that are delivered to PD in meristems.

The presence of some ER-luminal proteins and GPI-anchored proteins in the LB fraction appears puzzling. A possible explanation is that they might reside inside a subset of LBs. Most LBs are formed at the cytoplasmic leaflet of the ER, which then determines the composition of the monolayer and its associated proteins (Guo et al., 2009; Walther and Farese, 2009). Alternatively, an entire oil lens may be cut out from the ER, resulting in a bicelle LB, in which the monolayer is derived from both ER leaflets (Ploegh, 2007). Such LBs possess proteins that reside on both sites of the ER membrane and include luminal ER-anchored proteins. In addition, during “vesicular budding” (Walther and Farese, 2009), minute cytoplasmic bilayer vesicles are produced and tethered to ER, while neutral lipids are imported into the bilayer by a lipid shuttle. As a result, the growing LB can contain a minuscule aqueous inclusion inside the remnants of the luminal ER leaflet, as also observed in birch meristems (Rinne and van der Schoot, 2004). In the last two cases, LBs can contain luminal ER proteins, for example, calnexin and BIP (Table 1), which are indeed frequently found in LB fractions (Brasaemle et al., 2004; Liu et al., 2004; Umlauf et al., 2004; Ploegh, 2007). LBs might also recruit organellar proteins. For example, LBs interact with ER, the Golgi apparatus, and early and late endosomes (Beller et al., 2010), and the coatomer assemblies SAR1-COPII and GEF-ARF1-COPII are implicated in protein delivery to LBs (Soni et al., 2009). Moreover, Arf1/COP1 locates to LBs, probably directing ER proteins to bridges that connect the ER to LBs (Bartz et al., 2007; Beller et al., 2010; Wilfling et al., 2014; Gao and Goodman, 2015; Brocard et al., 2017; Huang et al., 2019). It is of interest, therefore, that COPII and SAR1/2, as well as COPI, GEF (BIG5), and ARF1, are present in the bud LB fraction (Table 1).

The current inventory of putative LB-associated proteins lays out a road map for future investigations into the shuttle function of LBs in plant meristems, serving cellular metabolism, organellar interaction, and uniquely for plants, transport to and through PD.

## Experimental Procedures

### Plant Materials and Sample Collection

Hybrid aspen (*Populus tremula* × *Populus tremuloides*) clone T89 plants were micro propagated, moved to a glass house, and grown under an 18-h long day (Veerabagu et al., 2020). When the plants reached a height of 80–100 cm, half of them were subjected to 10-h short days to induce dormant buds. We investigated meristems in three distinct phases: actively proliferating apices of growing long-day plants (APs), apices inside developing buds (DEBs, 3–4 weeks old), and dormant apices in completed terminal buds (DOBs, 6–9 weeks old).

*P. trichocarpa* OLE6-eGFP was overexpressed in *Arabidopsis thaliana* (Col-0), and homozygous lines were obtained as previously described by Veerabagu et al. (2020). Transgenic *Arabidopsis* PtOLE6-eGFP seeds were germinated on Whatman paper, wetted with 2 ml of 0.2% DMSO (control), 200 μM MG132, or 40 μM E64d (Deruyffelaere et al., 2015). Seeds were first subjected to 72 h of stratification at 4°C in the dark and later germinated under continuous light for 48–72 h as indicated at 25°C.

### Microscopy of SAMs and LBs

Shoot apices of growing plants (APs), developing buds (DEBs, 4 weeks) and dormant buds (DOBs, 8 weeks) were fixed overnight in 2% (v/v) glutaraldehyde and 3% (v/v) paraformaldehyde at 4°C in 100 mM phosphate citrate buffer (Rinne et al., 2001). Because of the density of the bud tissues, the infiltration into LR white resin (LRW) was done gradually by increasing the LRW concentration stepwise from 30 to 70%, and, finally, incubating 4–7 days in 100% LRW. The resin was polymerized at 55°C for 24 h. Samples were trimmed longitudinally, and 1 μm thick median sections were stained with 1% (w/v) aqueous toluidine blue for light microscopical observation (Leica DM6B, Leica Microsystems, Wetzlar, Germany) and imaging with a digital camera (Leica DMC4500, Leica Microsystems, Wetzlar, Germany). Subsequently, ultra-thin 80 nm sections were cut from samples and observed with a TEM (FEI Morgagni 268 FEI Company, Eindhoven, The Netherlands), which is electronically set to produce high-contrast LBs.

The number and sizes of LBs were assessed manually in median longitudinal TEM sections, restricted to two areas in the SAM and in the subjacent rib meristem/rib zone region (RM/RZ) (Figures 1A–C). Per photoperiod 2–3 replicate apices were used, and LB number and sizes were assessed in two TEM sections. As the chance to section through a LB increases with LB size (diameter), the numbers were corrected relative to DOB. In addition, we calculated the total number of LBs per average cell volume, considering section thickness and LB diameter, as explained in Supplementary Figure 1.

### RNA Extraction, Quantitative PCR (qPCR), and Cloning

Complementary DNA (cDNA) preparations derived from dormant buds were used as a template to amplify all the LDAPs. The LDAP entry clones were obtained by BP reaction in pDONR207 and verified by sequencing. The binary constructs for mRFP fusion protein expression under the control of the 35S promoter were obtained *via* LR reaction using the respective LDAP entry clones and the destination vector pB7WGR2 (Karimi et al., 2002). For qPCR analyses, we collected apices from growing long-day plants (APs), and from short-day plants with developing buds (DEBs, 3 weeks), and dormant buds (DOBs, 6 weeks). Three biological replicates, each containing pooled samples, were frozen in liquid nitrogen. RNA was extracted from 0.2–0.3 g of frozen samples and analyzed with quantitative qRT-PCR, as described before (Katyayini et al., 2019). All clones were constructed using the Gateway™ technology (Invitrogen, Waltham, MA, United States). The binary vector pK7FWG2 expressing PtOLE6-eGFP fusion protein was constructed as described previously by Veerabagu et al. (2020). cDNA preparations derived from dormant buds were used as a template to amplify all the LDAPs. The LDAP entry clones were obtained by BP reaction in pDONR207 and verified by sequencing. The binary constructs for mRFP fusion protein expression under the control of the 35S promoter were obtained *via* LR reaction using the respective LDAP entry clones and the destination vector pB7WGR2 (Karimi et al., 2002). Gene-specific primer sequences for qPCR analyses were designed using Primer3. The list of primers and genes used for qPCR and cloning are presented in Supplementary Tables 2, 3.

### Lipid Body Purification

Dormant buds were collected from short-day (9 weeks) exposed plants and used for LB purification and repeated in three independent experiments (Bio 1–3). Freshly harvested buds weighing 5–8 g were kept on ice until all the hard bud scales were carefully removed. The material was rinsed thrice in cold distilled water and then processed for LB extraction and purification. For LB purification, we used a previously described method (Jolivet et al., 2004) with two modifications. First, we deliberately omitted the NaCl ionic elution step to avoid discarding the loosely attached LB-associated proteins, which we aimed to identify. Second, proteins were precipitated using ice-cold methanol instead of hexane. Bio 1–2 received the prescribed 1X Tween20 wash, but Bio 3 was washed once more with Tween20 to increase stringency. The purity of the LB fraction was checked under light and DIC microscope to confirm absence of cellular contaminations. Total FAME measurement was carried out to assess the enrichment of fats in the LB fractions (O'Fallon et al., 2007). A fraction of the floating “fat pads” containing purified LBs was collected and inspected under the confocal microscope to confirm its purity and integrity (Supplementary Figure 4C). The fat pad, which was enriched with LBs (Supplementary Figure 4D), was further processed for protein precipitation (Veerabagu et al., 2020).

### Immunoblotting

To investigate the production of LDAP1 and OLEOSIN in *Populus* buds by immunodetection, axillary buds were obtained from different developmental zones of long and short-day plants, as indicated in Figure 6. Proteins were extracted using the 2x Laemmli buffer, and equal amounts of protein (10 mg) were loaded in each well. Western blot analyses were carried out using primary antibodies of *A. thaliana* anti-OLE1 polyclonal antibody (PhytoAB-PHY0954A) at a dilution ratio of 1:2000, *Hevea brasiliensis* anti-SRPP1 (LDAP1) monoclonal antibody (Abcam-ab138711, Abcam, Cambridge, United Kingdom) at a dilution ratio of 1:1000, and anti-eGFP monoclonal antibody (Thermo Fisher-MA1-952, Thermo-Fisher Scientific, Waltham, MA, United States) at a dilution ratio of 1:2000. Goat anti-rabbit IgG H&L (HRP) antibody phytoAB-PHY6000 at a dilution ratio of 1:5000 was used as a secondary antibody for OLE1. Goat anti-mouse IgG H&L (HRP) (Abcam-ab205719) at a dilution ratio of 1:5000 was used as a secondary antibody for both LDAP1 and eGFP antibodies. Immunoblot signals were detected with the Clarity Western ECL kit (Bio-Rad, Hercules, CA, United States) and imaged with a Chemidoc (Bio-Rad, Hercules, CA, United States) digital imager.

Degradation of OLEOSIN and inhibition of degradation were studied in imbibed seeds of transgenic PtOLE6-eGFP-expressing *Arabidopsis* seedlings (Figure 3). Proteins were extracted from imbibed PtOLE6-eGFP transgenic seeds and immunoblotted using an anti-eGFP monoclonal antibody (Thermo Fisher-MA1-952, Thermo-Fisher Scientific, Waltham, MA, United States) and detected in Western blots as described above.

### Protein Extraction

In short, the LB pellets were rinsed with methanol and air-dried. Subsequently, proteins were extracted in appropriate volumes of 2x Laemmli buffer. The third biological replicate received an additional wash, which further purified the sample. The extracted proteins were loaded on a 10% Bio-Rad mini protein gel (Bio-Rad, Hercules, CA, United States) and allowed to run ~1.5 cm into the gel. The protein gel was stained with Coomassie Brilliant Blue (MilliporeSigma, Burligton, United States) for an hour and destained overnight. The region of the gel showing stained proteins was isolated and excised into six fractions. The gel pieces were reduced (DTT) and alkylated (iodoacetamide) before overnight digestion with trypsin. After digestion, the peptides were extracted from the gel by sonication in a 0.1% TFA solution and cleaned up by a reversed-phase (C18) spin-tip procedure. Eluted peptides were dried and re-dissolved in a loading solution (2% acetonitrile, 0.05% TFA in MilliQ water). Samples were analyzed by LC-MS/MS, with Ultimate™ 3000 RSLC nano system coupled with Q Exactive hybrid quadrupole-orbitrap (Thermo-Fisher Scientific, Waltham, MA, United States). Thermo raw files were converted using the msfileconvert module of the Proteowizard (v 3.0.7076) software suite. All MS/MS samples were analyzed using Mascot (Matrix Science, London, UK; version 2.6.1). The mascot was set up to search the *P. trichocarpa* database (txid_3694, 53583 entries). The mascot was searched with a fragment ion mass tolerance of 0.02 Da and a parent ion tolerance of 10.0 PPM. Carbamidomethylation of cysteine was specified in Mascot as a fixed modification. Deamidation of asparagine and glutamine, oxidation of methionine, and acetylation of the n-terminus were specified in the Mascot as variable modifications.

### Criteria for Protein Identification

Scaffold (version Scaffold 4.9., Proteome Software Inc., Portland, OR, United States) was used to validate MS/MS-based peptide and protein identifications. Peptide identifications were accepted if they could be established at >95% probability by the Scaffold Local FDR algorithm. Protein identifications were accepted if they could be established at >99% probability and contained at least two identified peptides. Protein probabilities were assigned by the Protein Prophet algorithm (Nesvizhskii et al., 2003). Proteins that contained similar peptides and could not be differentiated based on MS/MS analyses alone were grouped to satisfy the principles of parsimony. Proteins were considered identified only if in all three biological replicates they contained at least two unique peptides. The mass spectrometry proteomics data have been deposited to the ProteomeXchange Consortium *via* the PRIDE^*^ (Perez-Riverol et al., 2019) partner repository with the dataset identifier PXD020099 and 10.6019/PXD020099. Gene ontology information was retrieved using The Arabidopsis Information Resource (TAIR) (Berardini et al., 2015) and The Plant Genome Integrative Explorer Resource (PlantGenIE) (Sundell et al., 2015). The complete list of *Populus* lipid body proteins identified in all the three biological replicates and the proteins identified in individual biological replicates are presented in Supplementary Table 1. The *P. trichocarpa* LB proteins identified in this study were checked for their relative protein abundance of different cellular compartments using the Multiple Marker Abundance Profiling (MMAP) tool from the SUBA toolbox and presented in Supplementary Figure 10.

### Protein *in situ* Localization

The corresponding binary constructs were transformed into the *A. tumefaciens* strain GV3101 pMP90 and further infiltrated into *N. benthamiana* leaves (Schutze et al., 2009) together with the p19 protein from tomato bushy stunt virus cloned in pBIN61 (Voinnet et al., 2000) to suppress gene silencing. After two days of infiltration, tobacco leaf epidermis was investigated with a Leica TCS SP5 CLSM (Leica Microsystems, Wetzlar, Germany), and eGFP (Veerabagu et al., 2020) and mRPF (Van Damme et al., 2004) fluorescence was recorded (eGFP: excitation/emission 488/500–540 nm; mRFP: excitation/emission 561/600–640 nm). The Leica Application Suite X software was used to arrange single optical sections of mRFP (in magenta), eGFP and the corresponding merged images. Degradation of PtOLE6-eGFP and inhibition of degradation were studied in seeds of transgenic PtOLE6-eGFP expressing Arabidopsis seedlings imbibed with 2 ml of.2% DMSO (control), 200 μM MG132, and 40μM E64d, and investigated with CLSM (Figure 3).

### Statistical Analyses and Bioinformatics

One-way ANOVA followed by Fisher's and Tukey's multiple comparisons tests were performed using GraphPad Prism Version 8.0.0 (www.graphpad.com). The MEGA6 program (http://www.megasoftware.net/) was used for ClustalW multiple sequence alignments and phylogenetic analysis, with maximum likelihood method and Poisson correction model. The *Populus* and *Arabidopsis* proteins of the phylogenetic analyses are presented in Supplementary Figures 2, 3, 6–9.

## Data Availability Statement

The original contributions presented in the study are publicly available. This data can be found here: https://www.ebi.ac.uk/pride/archive/projects/PXD020099.

## Author Contributions

MV, PR, LP, and CvdS designed the study. MV, PR, LP, and MS performed the experiments. MV and PR analyzed the data. MV, CvdS, and PR interpreted the data and wrote the manuscript. All authors contributed to the article and approved the submitted version.

## Conflict of Interest

The authors declare that the research was conducted in the absence of any commercial or financial relationships that could be construed as a potential conflict of interest.
