# Supplementary material for: Lipid Body Dynamics in Shoot Meristems: Production, Enlargement, and Putative Organellar Interactions and Plasmodesmal Targeting
Source: Front Plant Sci. 2021 Jul 21;12:674031. doi: 10.3389/fpls.2021.674031 (PMC8335594; doi:10.3389/fpls.2021.674031)
Supplement: Supplementary file 1 [file Image_1.pdf]

A

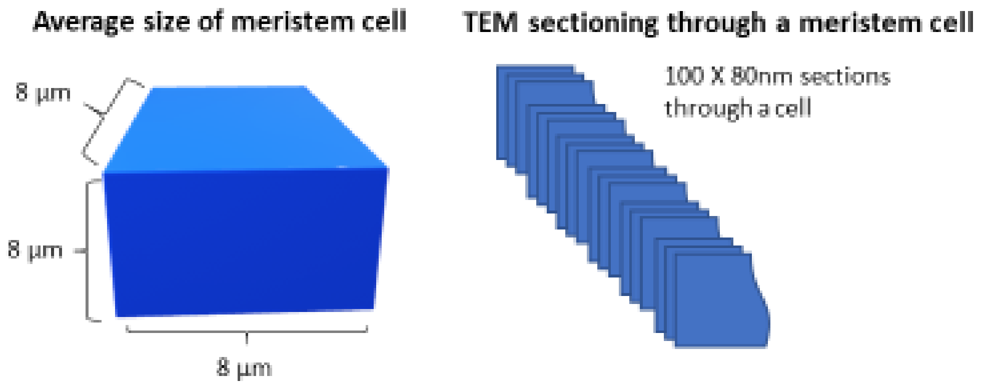

B

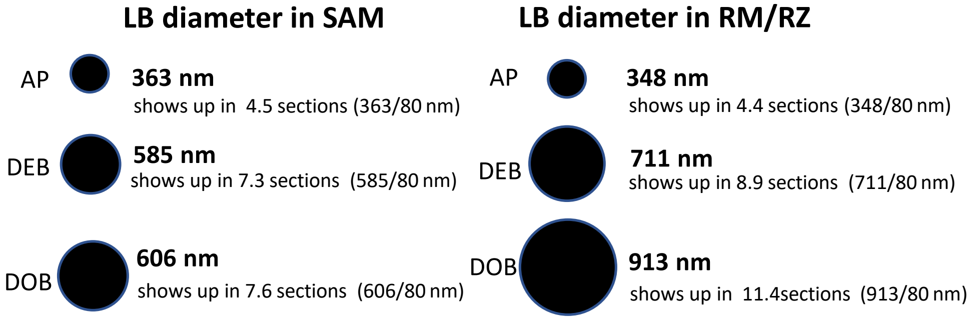

C

Calculated LB number /cell volume

Cell position	LB/cell /section	Sections through cell	LB diameter /80 nm	LBs/cell volume	Total LBs/cell
AP SAM	1.81	100	4.5	40.2	34.2
DEB SAM	3.75	100	7.3	51.4	43.7
DOB SAM	4.37	100	7.6	57.5	48.9
AP RM/RZ	0.80	100	4.4	18.2	15.5
DEB RM/RZ	3.50	100	8.9	39.3	33.4
DOB RM/RZ	4.32	100	11.4	37.9	32.2

D

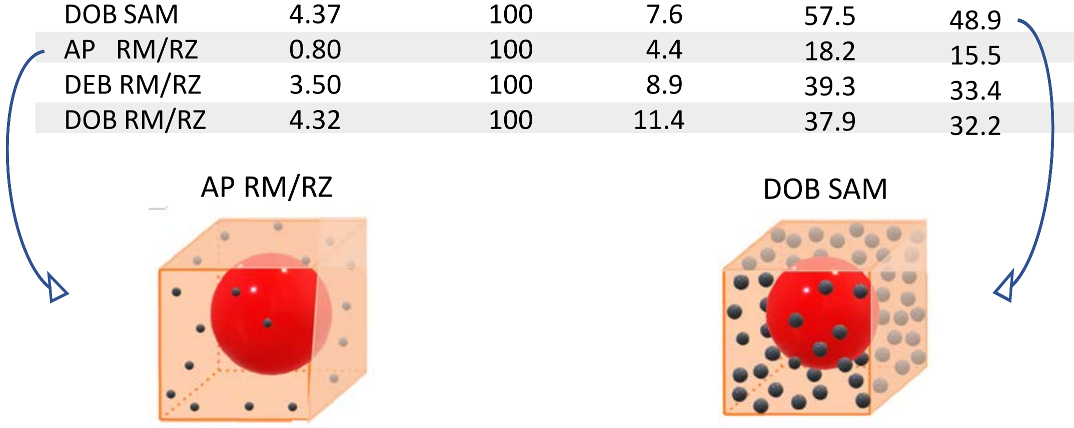

Figure S1. Calculation of lipid body (LB) number in cell volume from 80nm TEM sections. **(A)** The average size of a meristem cell is 8µm^3 amounting to 100 sections with 80nm thickness. **(B)** LB size affects the likelihood in which it will show up in a section. For example, LBs in a rib meristem (RM/RZ) of long day plants are on average ∅ 348nm and will show up in 4.4 successive sections (=348/80), whereas large LBs, e.g. in the SAM of DOB are ∅ 606nm, and present in 7.6 sections. **(C)** The observed LB number per cell per section (bars in Figure 1D) was multiplied by the number of sections (100) and divided by the LB size/80 nm to estimate their number per cell volume. Based on control analysis of 100 meristem cells (long day apices) real LB numbers are estimated to be 15% lower due to the presence of the nucleus and organelles (column Total LBs/cell). **(D)** Schematic representation of the lowest and highest LB number calculated per cell volume. AP, shoot apex of long day plant; DEB, developing bud in 3-4 weeks in short days; DOB, dormant bud in 6-9 weeks in short day.
