# Supplementary material for: Lipid Body Dynamics in Shoot Meristems: Production, Enlargement, and Putative Organellar Interactions and Plasmodesmal Targeting
Source: Front Plant Sci. 2021 Jul 21;12:674031. doi: 10.3389/fpls.2021.674031 (PMC8335594; doi:10.3389/fpls.2021.674031)
Supplement: Supplementary file 2 [file Image_2.pdf]

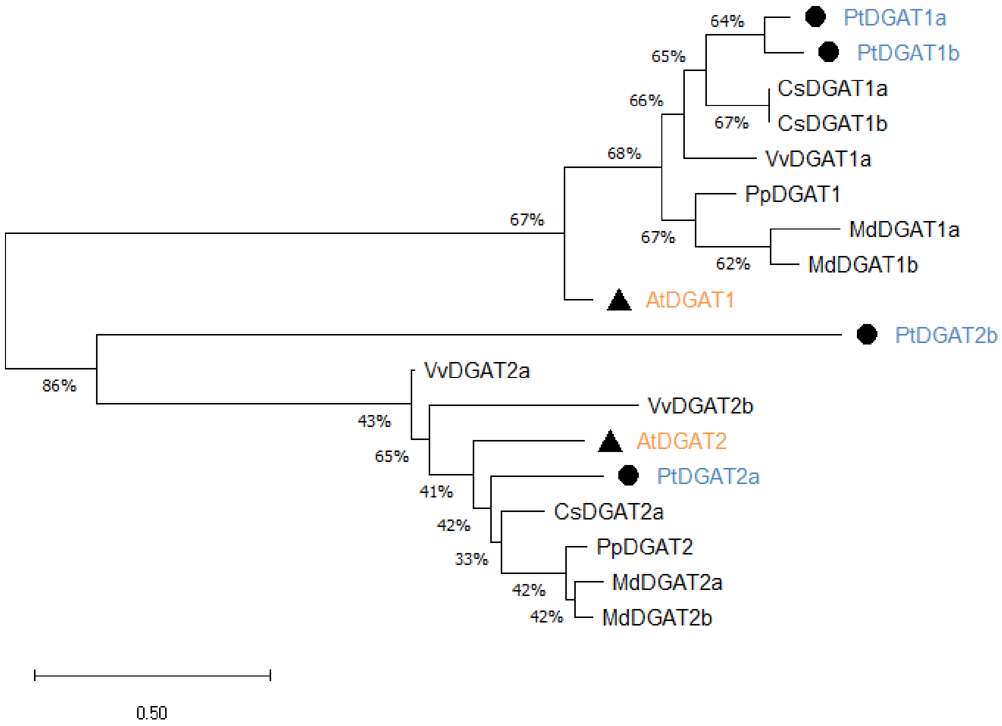

Figure S2. Phylogenetic analysis of Diacylglycerol O-Acyltransferases 1 and 6 (DGAT1 and DGAT6). The *Arabidopsis thaliana* sequence homologues were dentified by protein BLAST search and sequences were retrieved from the plant genomics resource database (Goodstein *et al.*, 2012; http//www.phytozome.net/). The amino acid sequence alignment was performed, and a phylogenetic tree was constructed using the MEGA-X program with the maximum likelyhood method and the Poisson correction model. The proteins used in this phylogenetic analysis were: *Arabidopsis thaliana* AtDGAT1 (AT2G19450), AtDGAT2 (AT3G51520); *Populus trichocarpa* PtDGAT1a (Potri.018G066100), PtDGAT1b (Potri.006G147600), PtDGAT2a (Potri.011G145900), PtDGAT2b (Potri.001G324200); *Vitis vinifera* VvDGAT1a (GSVIVT01015174001), VvDGAT2a (GSVIVT01026423001), VvDGAT2b (GSVIVT01026421001); *Prunus persica* PpDGAT1 (Prupe.7G206100), PpDGAT2 (Prupe.7G188100); *Citrus sinensis* CsDGAT1a (orange1.1g010418m), CsDGAT1b (orange1.1g010468m), CsDGAT2a (orange1.1g025152m); *Malus domestics* MdDGAT1a (MDP0000314313), MdDGAT1b (MDP0000297929), MdDGAT2a (MDP0000131182), MdDGAT2b (MDP0000137428). The percent of data coverage for internal nodes are displayed. AtDGATs (▲); PtDGATs (●).
