# Supplementary material for: Lipid Body Dynamics in Shoot Meristems: Production, Enlargement, and Putative Organellar Interactions and Plasmodesmal Targeting
Source: Front Plant Sci. 2021 Jul 21;12:674031. doi: 10.3389/fpls.2021.674031 (PMC8335594; doi:10.3389/fpls.2021.674031)
Supplement: Supplementary file 3 [file Image_3.pdf]

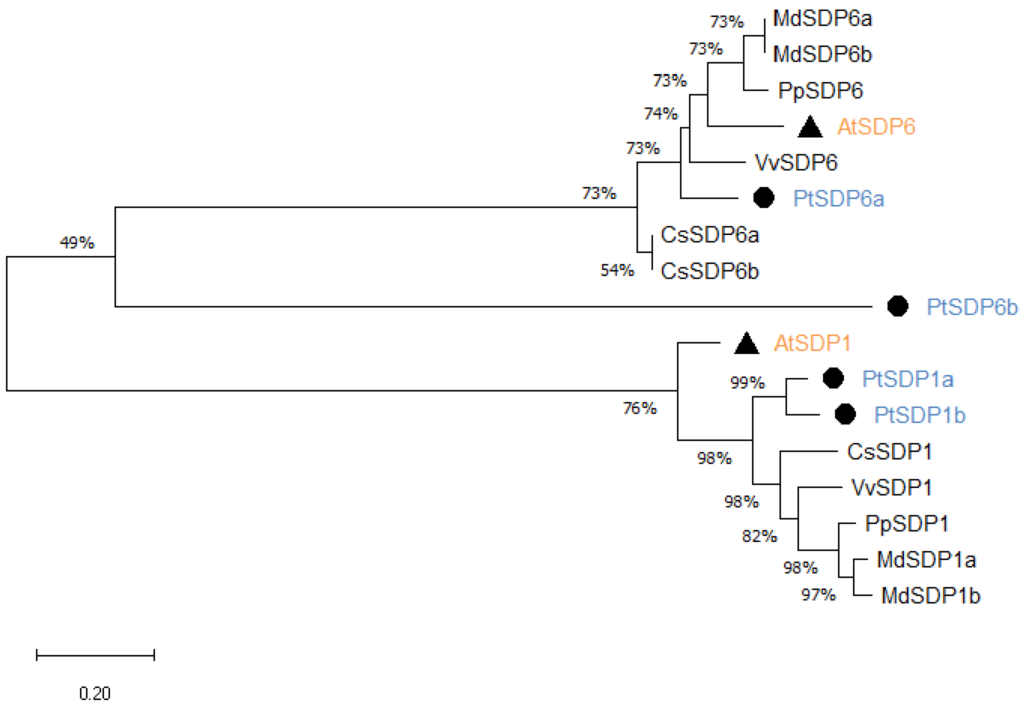

Figure S3. Phylogenetic analysis of Sugar Dependent Triacylglycerol Lipase 1 and 6 (SDP1 and SDP6). The *Arabidopsis thaliana* sequence homologues were identified by protein BLAST search and sequences were retrieved from the plant genomics resource database (Goodstein *et al.*, 2012; http//www.phytozome.net/). The amino acid sequence alignment was performed, and a phylogenetic tree was constructed using the MEGA-X program with the maximum likelyhood method and the Poisson correction model. The proteins used in this phylogenetic analysis were: *Arabidopsis thaliana* AtSDP1 (AT5G04040), AtSDP6 (AT3G10370); *Populus trichocarpa* PtSDP1a (Potri.006G043800), PtSDP1b (Potri.016G041000), PtSDP6a (Potri.010G226700), PtSDP6b (Potri.006G026100); *Vitis vinifera* VvSDP1 (GSVIVT01033566001), VvSDP6 (GSVIVT01016222001); *Prunus persica* PpSDP1 (Prupe.7G047400), PpSDP6 (Prupe.2G209000); *Citrus sinensis* CsSDP1 (orange1.1g003105m), CsSDP6a (orange1.1g006891m), CsSDP6b (orange1.1g012358m); *Malus domestics* MdSDP1a (MDP0000296428), MdDGAT1b (MDP0000136922), MdSDP6a (MDP0000293613), MdSDP6b (MDP0000253362). The percent of data coverage for internal nodes are displayed. AtSDPs (▲); PtSDPs (●).
