# Supplementary material for: Lipid Body Dynamics in Shoot Meristems: Production, Enlargement, and Putative Organellar Interactions and Plasmodesmal Targeting
Source: Front Plant Sci. 2021 Jul 21;12:674031. doi: 10.3389/fpls.2021.674031 (PMC8335594; doi:10.3389/fpls.2021.674031)
Supplement: Supplementary file 4 [file Image_4.pdf]

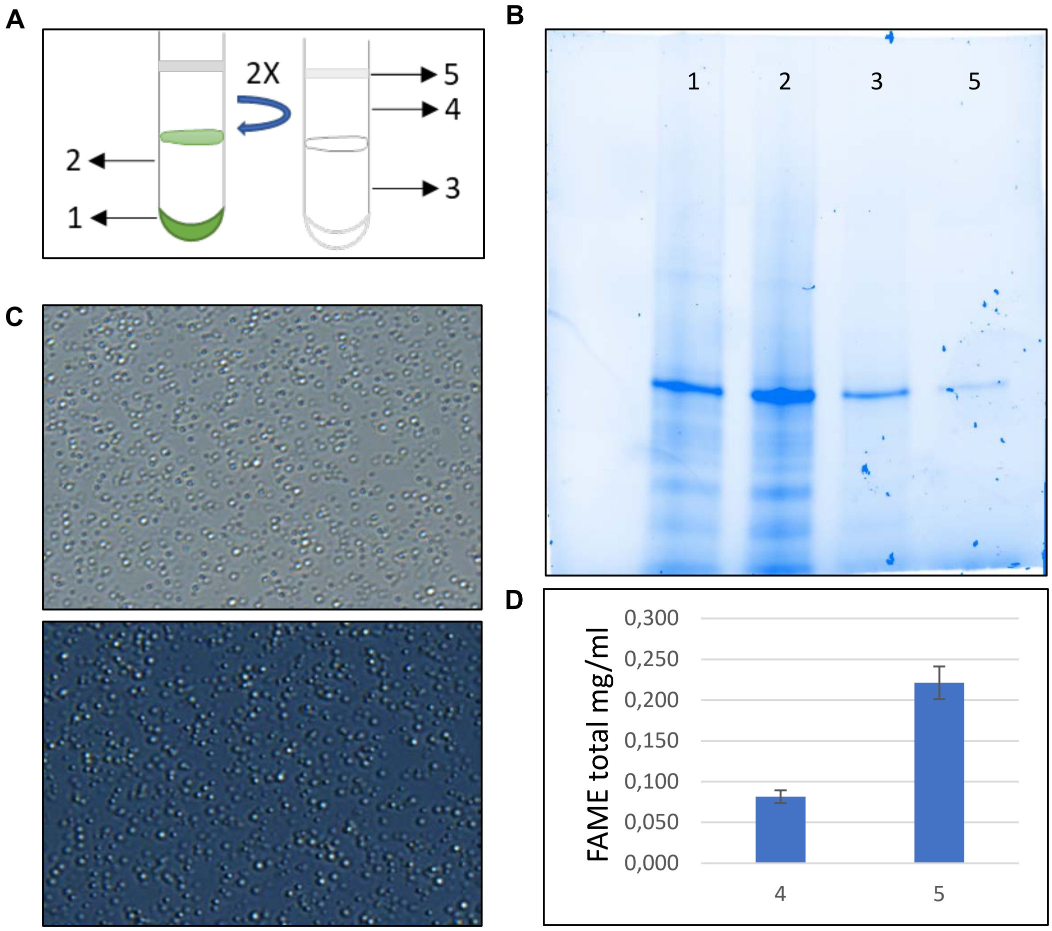

Figure S4. Isolation and enrichment of lipid body (LB) from dormant buds of Populus. **(A)** A schematic representation of fractions used for SDS-PAGE, microscopic and lipid analyses **(B-D)**. Abbreviations: 1. Pellete; 2. Bottom layer after first centrifugation in phosphate buffer with 0.6M Sucrose; 3. Bottom layer after fourth centrifugation in phosphate buffer with 0.2M sucrose and 0.1% Tween 20; 4. Top layer after fourth centrifugation in 10 mM phosphate buffer; 5. Final purified LB fraction. **(B)** Coomassie stained SDS-PAGE of total protein from different fractions, as indicated in **A**. **(C)** Bright field (top) and Differential Interference Contrast (bottom) microscopic images showing the enrichment of LBs in the final LB fraction, 40x magnification. **(D)** Total Fatty Acid Methyl Ester (FAME) contents of two different fractions (4 and 5) were quantified by gas chromatography, confirming the enrichment of fats in the LB fraction.
