# Supplementary material for: Lipid Body Dynamics in Shoot Meristems: Production, Enlargement, and Putative Organellar Interactions and Plasmodesmal Targeting
Source: Front Plant Sci. 2021 Jul 21;12:674031. doi: 10.3389/fpls.2021.674031 (PMC8335594; doi:10.3389/fpls.2021.674031)
Supplement: Supplementary file 5 [file Image_5.pdf]

A

BDM-PUB of PtOLE6- Potri.006G234900:

MPDRSRPMSRYEPSSGQPTSRKAVKFMTAGTIGAALLVLSGLTLTGTVIALVVATPVLVLSSPILVPAAIV
VFLVASGFFFSSGCGLAAIMVSLWIYNYVTGKHPPGADKLDYAGGRIAETAKDMKDRAKECGQNVRQ
KVQESSHTQTS

Peptide	Position	Score	Threshold
SGQPTSRKAVKFMTA	22	2.27	0.3
PTSRKAVKFMTAGTI	25	1.42	0.3
KHPPGADKLDYAGGR	110	2.03	0.3
GRIAETAKDMKDRAK	123	1.65	0.3
AETAKDMKDRAKECG	126	2.07	0.3
KDMKDRAKECGQNVR	130	0.80	0.3

B

```
AtOLE-S4_OLE2_      MADTHRVDRTDRHFQFQSPYEGGRGQGQYEGDRGYGGGGYKSMMPESGPSSTQVLSLLIG 60
AtOLE-S2_OLE3_      MANVDRDRRVHVDRTDKRVHQPNYEDDVGFG--GYGGYGAGSDYKSRGPSTNQILALIAG 58
AtOLE-S1_OLE4_      ----MADVRTHSHQLQVHPQRQHEG------------GIKVLYPQSGPSSTQVLAVFVG 43
AtOLE-S3_OLE1_      MADTARGTHHDIIGRDQYPMMGRDRDQ-----------YQMSGRGSDYSKSRQIAKAATA 49
PtOLE6              -----------------MPDRSRPMSR-----------YEPS---SGQPTSRKAVKFMTA 29
                                                                 .  ..: :      .

AtOLE-S4_OLE2_      VPVVGSLLALAGLLLAGSVIGLMVALPLFLLFSPVIVPAALTIGLAMTGFLASGMFGLTG 120
AtOLE-S2_OLE3_      VPIGGTLLTLAGLTLAGSVIGLLVSIPLFLLFSPVIVPAALTIGLAVTGILASGLFGLTG 118
AtOLE-S1_OLE4_      VPIGGTLLTIAGLTLAGSVIGLMLAFPLFLIFSPVIVPAAFVIGLAMTGFLASGAIGLTG 103
AtOLE-S3_OLE1_      VTAGGSLLVLSSLTLVGTVIALTVATPLLVIFSPILVPALITVALLITGFLSSGGFGIAA 109
PtOLE6              GTIGAALLVLSGLTLTGTVIALVVATPVLVLSSPILVPAAIVVFLVASGFFFSSGCGLAA 89
                     .  .:**.::.* *.*:**.* :: *:::: **::*** :.: *  :*:: *.  *::.

AtOLE-S4_OLE2_      LSSISWVMNYLRGTRRTVPEQLEYAKRRMADAVGYAGQKGKEMGQHVQNKAQDVKQYDIS 180
AtOLE-S2_OLE3_      LSSVSWVLNYLRGTSDTVPEQLDYAKRRMADAVGYAGMKGKEMGQYVQDKAHEARETEFM 178
AtOLE-S1_OLE4_      LSSMSWVLNYIRRAGQHIPEELEEAKHRLADMAEYVGQRTKDAGQTIEDKAHDVREAKTF 163
AtOLE-S3_OLE1_      ITVFSWIYKYATGEHPQGSDKLDSARMKLGSKAQDLKDRAQYYGQQHTGGEHDRDRTRGG 169
PtOLE6              IMVSLWIYNYVTGKHPPGADKLDYAGGRIAETAKDMKDRAKECGQNVRQKVQESSHTQTS 149
                    :    *: :*        .::*: *  ::.. .     : :  **      ::  .

AtOLE-S4_OLE2_      KPHDTTTKGHETQGRTTAA-
AtOLE-S2_OLE3_      TETHEPGKARRGS-------
AtOLE-S1_OLE4_      DVRDRDTTKGTHNVRDTKTT
AtOLE-S3_OLE1_      QHTT----------------
PtOLE6              --------------------
```

Protein Name	Major Ubiquitinated peptide	Minor Ubiquitinated peptide
AtOLE1	K141	K130
AtOLE2	K159	K175
AtOLE3	K159	K157
AtOLE4	K144	K153

Figure S5. Predicted ubiquitinated PtOLE6 peptides. Alignment of PtOLE6 peptides with the known Arabidopsis OLEOSIN's ubiquitinated peptides. **(A)** Prediction of *Populus trichocarpa* OLEOSIN6 ubiquitinated peptides with Bayesian Discriminant algorithm Method (BDM-PUB) (http://bdmpub.biocuckoo.org/index.php). **(B)** PtOLE6 aligned with the known ubiquitination sites of AtOLE1-OLE4 (Deruyffelaere et al,. 2015).
