# Supplementary material for: Lipid Body Dynamics in Shoot Meristems: Production, Enlargement, and Putative Organellar Interactions and Plasmodesmal Targeting
Source: Front Plant Sci. 2021 Jul 21;12:674031. doi: 10.3389/fpls.2021.674031 (PMC8335594; doi:10.3389/fpls.2021.674031)
Supplement: Supplementary file 6 [file Image_6.pdf]

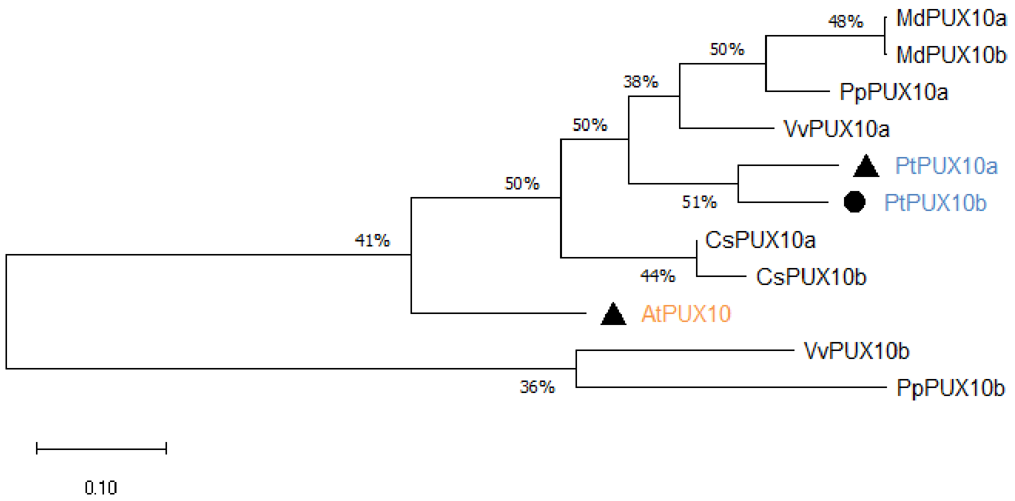

Figure S6. Phylogenetic analysis of Plant UBX-domain Containing Protein 10 (PUX10). The *Arabidopsis thaliana* sequence homologues were identified by protein BLAST search and sequences were retrieved from the plant genomics resource database (Goodstein *et al.*, 2012; http//www.phytozome.net/). The aminoacid sequence alignment was performed, and a phylogenetic tree was constructed using the MEGA-X program with the maximum likelyhood method and the Poisson correction model. The proteins used in this phylogenetic analysis were: *Arabidopsis thaliana* AtPUX10 (AT4G10790); *Populus trichocarpa* PtPUX10a (Potri.001G085600), PtPUX10b (Potri.003G145200); *Vitis vinifera* VvPUX10a (GSVIVT01019629001), VvPUX10b (GSVIVT01007694001); *Prunus persica* PpPUX10a (Prupe.5G127400), PpPUX10b (Prupe.5G170100); *Citrus sinensis* CsPUX10a (orange1.1g012346m), CsPUX10b (orange1.1g015165m); *Malus domestics* MdPUX10a (MDP0000269352), MdPUX10b (MDP0000638064). The percent of data coverage for internal nodes are displayed. AtPUX10 (▲); PtPUX10s (●).
