# Supplementary material for: Lipid Body Dynamics in Shoot Meristems: Production, Enlargement, and Putative Organellar Interactions and Plasmodesmal Targeting
Source: Front Plant Sci. 2021 Jul 21;12:674031. doi: 10.3389/fpls.2021.674031 (PMC8335594; doi:10.3389/fpls.2021.674031)
Supplement: Supplementary file 7 [file Image_7.pdf]

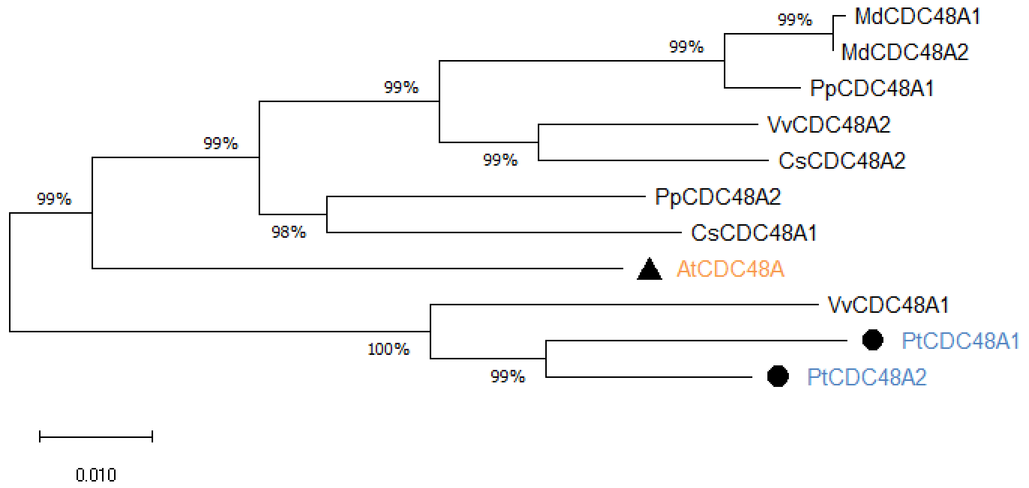

Figure S7. Phylogenetic analysis of Cell Division Cycle 48A (CDC48A). The *Arabidopsis thaliana* sequence homologues were identified by protein BLAST search and sequences were retrieved from the plant genomics resource database (Goodstein *et al.*, 2012; http//www.phytozome.net/). The aminoacid sequence alignment was performed, and a phylogenetic tree was constructed using the MEGA-X program with the maximum likelyhood method and the Poisson correction model. The proteins used in this phylogenetic analysis were: *Arabidopsis thaliana* AtCDC48A (AT3G09840); *Populus trichocarpa* PtCDC48A1 (Potri.012G088200), PtCDC48A2 (Potri.015G080600); *Vitis vinifera* VvCDC48A1 (GSVIVT01007689001), VvCDC48A2 (GSVIVT01025723001); *Prunus persica* PpCDC48A1 (Prupe.6G233700), PpCDC48A2 (Prupe.6G107800); *Citrus sinensis* CsCDC48A1 (orange1.1g003620m), CsCDC48A2 (orange1.1g003623m); *Malus domestics* MdCDC48A1 (MDP0000175405), MdCDC48A2 (MDP0000181072). The percent of data coverage for internal nodes are displayed. AtCDC48A (▲); PtCDC48As (●).
