# Supplementary material for: Lipid Body Dynamics in Shoot Meristems: Production, Enlargement, and Putative Organellar Interactions and Plasmodesmal Targeting
Source: Front Plant Sci. 2021 Jul 21;12:674031. doi: 10.3389/fpls.2021.674031 (PMC8335594; doi:10.3389/fpls.2021.674031)
Supplement: Supplementary file 8 [file Image_8.pdf]

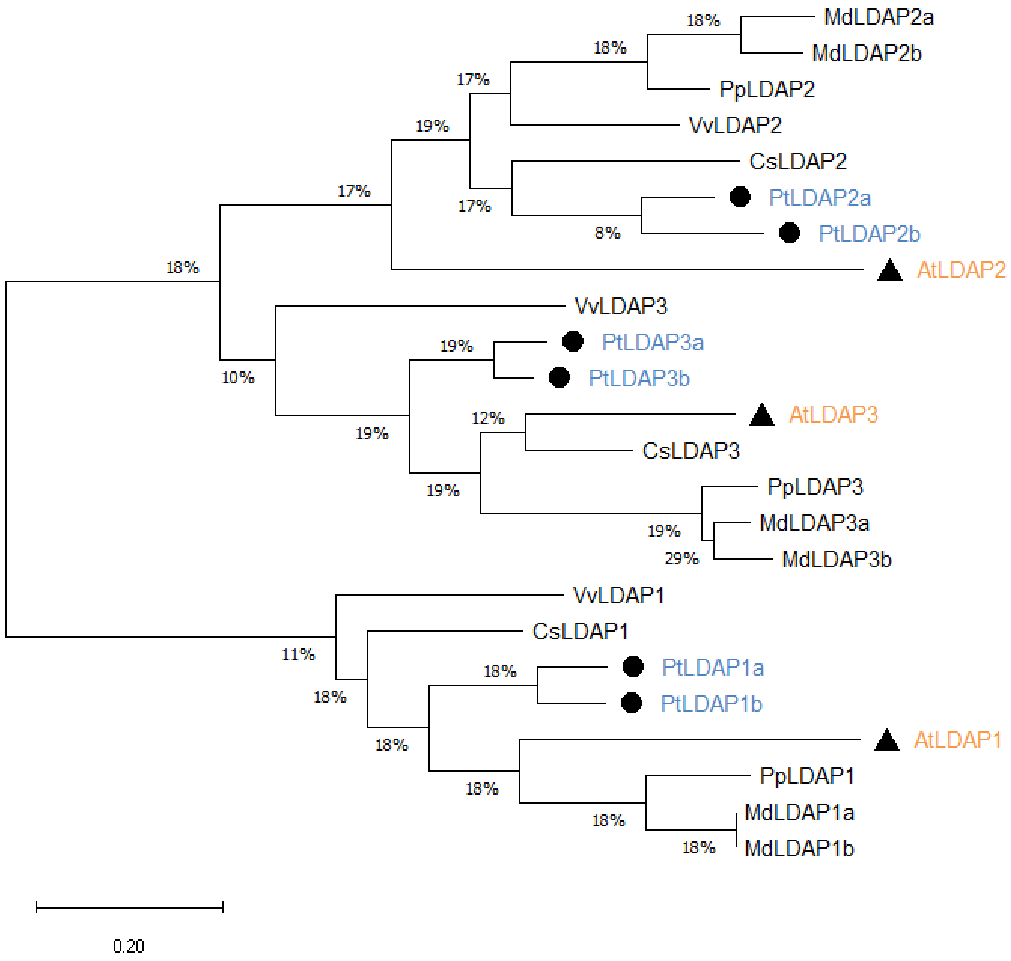

Figure S8. Phylogenetic analysis of Lipid Droplet Associated Proteins (LDAPs). The *Arabidopsis thaliana* sequence homologues were identified by protein BLAST search and sequences were retrieved from the plant genomics resource database (Goodstein *et al.*, 2012; http//www.phytozome.net/). The aminoacid sequence alignment was performed, and a phylogenetic tree was constructed using the MEGA-X program with the maximum likelyhood method and the Poisson correction model. The proteins used in this phylogenetic analysis were: *Arabidopsis thaliana* AtLDAP1 (AT1G67360), AtLDAP2 (AT2G47780), AtLDAP3 (AT3G05500); *Populus trichocarpa* PtLDAP1a (Potri.001G055300), PtLDAP1b (Potri.003G173100), PtLDAP2a (Potri.002G206000), PtLDAP2b (potri.014G131100), PtLDAP3a (Potri.005G025700), PtLDAP3b (Potri.013G017300); *Vitis vinifera* VvLDAP1 (GSVIVT01010480001), VvLDAP2 (GSVIVT01027980001), VvLDAP3(GSVIVT01031148001); *Prunus persica* PpLDAP1 (Prupe.1G226000), PpLDAP2 (Prupe.8G258700), PpLDAP3 (Prupe.6G321100); *Citrus sinensis* CsLDAP1 (orange1.1g026954m), CsLDAP2 (orange1.1g027165m), CsLDAP3 (orange1.1g036810m); *Malus domestics* MdLDAP1a (MDP0000145643), MdLDAP1b (MDP0000178529), MdLDAP2a (MDP0000267643), MdLDAP2b (MDP0000574987), MdLDAP3a (MDP0000557646), MdLDAP3b (MDP0000608906). The percent of data coverage for internal nodes are displayed. AtLDAPs (▲); PtLDAPs (●).
