# Supplementary material for: Lipid Body Dynamics in Shoot Meristems: Production, Enlargement, and Putative Organellar Interactions and Plasmodesmal Targeting
Source: Front Plant Sci. 2021 Jul 21;12:674031. doi: 10.3389/fpls.2021.674031 (PMC8335594; doi:10.3389/fpls.2021.674031)
Supplement: Supplementary file 9 [file Image_9.pdf]

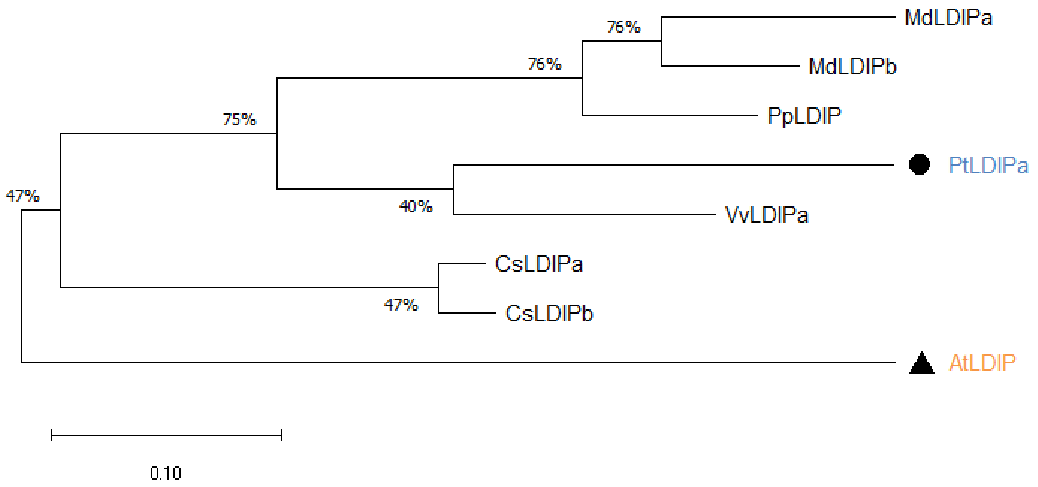

Figure S9. Phylogenetic analysis of Lipid Droplet Associated Protein Interacting Protein (LDIP). The *Arabidopsis thaliana* sequence homologues were identified by protein BLAST search and sequences were retrieved from the plant genomics resource database (Goodstein *et al.*, 2012; http//www.phytozome.net/). The aminoacid sequence alignment was performed, and a phylogenetic tree was constructed using the MEGA-X program with the maximum likelyhood method and the Poisson correction model. The proteins used in this phylogenetic analysis were *Arabidopsis thaliana* AtLDIP (AT5G16550); *Populus trichocarpa* PtLDIP (Potri.004G082300); *Vitis vinifera* VvLDIP (GSVIVT01026318001); *Prunus persica* PpLDIP (Prupe.3G163400); *Citrus sinensis* CsLDIPa (orange1.1g032604m), CsLDIPb (orange1.1g032586m); *Malus domestics* MdLDIPa (MDP0000292886), MdLDIPb (MDP0000289556). The percent of data coverage for internal nodes are displayed. AtLDIP (▲); PtLDIPs (●).
