# Supplementary material for: Lipid Body Dynamics in Shoot Meristems: Production, Enlargement, and Putative Organellar Interactions and Plasmodesmal Targeting
Source: Front Plant Sci. 2021 Jul 21;12:674031. doi: 10.3389/fpls.2021.674031 (PMC8335594; doi:10.3389/fpls.2021.674031)
Supplement: Supplementary file 10 [file Image_10.pdf]

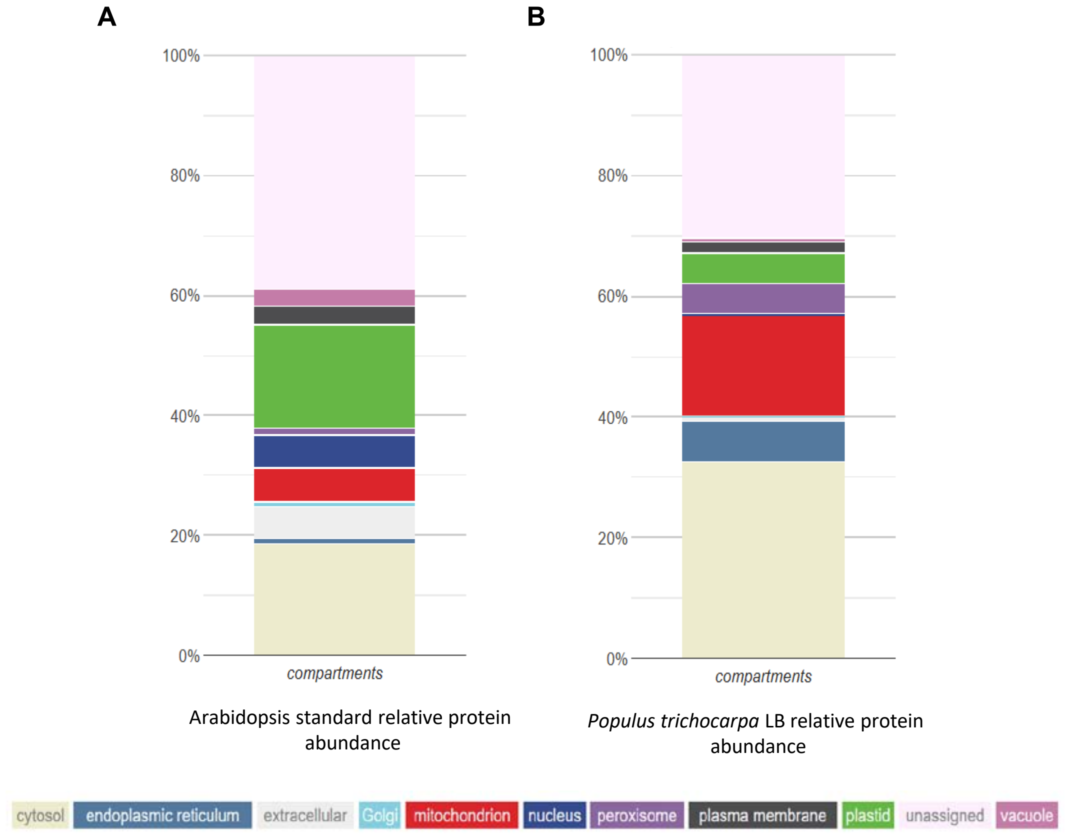


**Figure S10**. Estimation of the relative protein abundance of different cellular compartments. The Multiple Marker Abundance Profiling (MMAP) tool from the SUBA Toolbox was used with default settings (https://suba.live/toolbox-app.html). Relative protein abundance for standard Arabidopsis proteins **(A)** compared with Populus bud LB proteins **(B)** identified in our study. The closest Arabidopsis homolog of each Populus gene was used for the estimation.
