# Supplementary material for: Lipid Body Dynamics in Shoot Meristems: Production, Enlargement, and Putative Organellar Interactions and Plasmodesmal Targeting
Source: Front Plant Sci. 2021 Jul 21;12:674031. doi: 10.3389/fpls.2021.674031 (PMC8335594; doi:10.3389/fpls.2021.674031)
Supplement: Supplementary file 12 [file Table_2.pdf]

Table S2. *P. trichocarpa* genes, identifiers and primer pairs used for qPCR analysis.

Populus trichocarpa				
**Protein**	**Gene abbreviation**	**Locus Name V3.0**	**Forward 5' - 3'**	**Reverse 5' - 3'**
**TAG biosynthesis Genes**				
Diacylglycerol O-acyltransferase 1a	DGAT1a	Potri.018G066100	TTTCACTATATGCCCTGCAC	GAAGCCGAGTCCGTGACTGT
Diacylglycerol O-acyltransferase 1b	DGAT1b	Potri.006G147600	TCAACGGCAACAACATCATC	ATCCGATCGATTCTCATTCG
**Sugar Dependent TAG Lipases**				
Sugar Dependent 1a	SDP1a	Potri.006G043800	CAAGGGTAGGCTTCAAGTGC	GAAACGCTCCAAGTGAAGCA
Sugar Dependent 1b	SDP1b	Potri.016G041000	CCAATAGGGTCCACGATAAGGA	AAGGGATTTGATGGGGAGGG
Sugar Dependent 6a	SDP6a	Potri.010G226700	TTGTGCCCGGAATGAGTACT	GTCCCACTTGTGTTCAGCAG
Sugar Dependent 6b	SDP6b	Potri.006G026100	TGTGAGGGGAAGAGAATTGC	CCCATTTTGAGTTCCACGAT
**Plant UBX-domain containing proteins**				
Plant UBX domain-containing protein 10a	PUX10a	Potri.001G085600	CAGTGGGGTTTGGATTATGG	CGCAGCAGAAACAGTTACCA
Plant UBX domain-containing protein 10b	PUX10b	Potri.003G145200	CAGATCCTCCAAGCCCATAA	GATGAAATGGGTGGTTCGAC
**Cell division cycle proteins**				
Cell division control protein 48A1	CDC48A1	Potri.012G088200	AAGGCATTTGAGGAAGCTGA	GTCAATGCTGTTGGGCCTAT
Cell division control protein 48A2	CDC48A2	Potri.015G080600	TTGACATTGGTGTGCCAGAT	AAGAGCAGCCTCGGTACAAA
**LD- Associated proteins**				
Lipid droplet associated protein 1a	LDAP1a	Potri.001G055300	GAGGATAGCTGCGATTCAGG	CCTCAACAGTTCCAACAGCA
Lipid droplet associated protein 1b	LDAP1b	Potri.003G173100	AGATGGAGCAACAGCCAAGT	TCCTCCTGCTTGAAACTCGT
Lipid droplet associated protein 2a	LDAP2a	Potri.002G206000	TCCACTCTTCCCTCAAGTGG	GGGATCAACGGCAGGTATAA
Lipid droplet associated protein 2b	LDAP2b	potri.014G131100	CGAAGGAAAATTCAGGTCCA	AGGGGACCCCATAAAACTTG
Lipid droplet associated protein 3a	LDAP3a	Potri.005G025700	CCGAACTGGCAAAATCTGTT	GGAAGAGTGGGAGCTGATTG
Lipid droplet associated protein 3b	LDAP3b	Potri.013G017300	AAGCAGGTGTCATTCCAAGC	GAGGCTGTTCCTTTCACACC
LDAP-Interacting protein a	LDIPa	Potri.004G082300	TGATCAATGGAGTGGAAGCA	GCTTCCTGGATCAGGGTACA
**Housekeeping gene**				
Actin	ACT	Potri.001G309500	CGATGCCGAGGATATTCAAC	ACCAGTGTGTCTTGGTCTACCC
