# Supplementary material for: Lipid Body Dynamics in Shoot Meristems: Production, Enlargement, and Putative Organellar Interactions and Plasmodesmal Targeting
Source: Front Plant Sci. 2021 Jul 21;12:674031. doi: 10.3389/fpls.2021.674031 (PMC8335594; doi:10.3389/fpls.2021.674031)
Supplement: Supplementary file 13 [file Table_3.pdf]

Table S3. Primer sequences used for cloning the *P.trichocarpa* LDAP ENTRY (pDON207) clones.

Genes	Forward 5'-3'	Reverse 5'-3'
LDAP1a	GGGG**ACAAGTTTGTACAAAAAAGCAGGCT**TAATGGAGGTAGAGAACAGCAAGAG	GGG G**ACCACTTTGTACAAGAAAGCTGGGT**CATCGGAGTCTGACGAATCCGAATC
LDAP1b	GGGG**ACAAGTTTGTACAAAAAAGCAGGCT**TAATGGGGATGATGGAGGTAGACAG	GGGG**ACCACTTTGTACAAGAAAGCTGGGT**CTCAGAGTCTGATGAACTCGAATCT
LDAP2a	GGGG**ACAAGTTTGTACAAAAAAGCAGGCT**TAATGGCGGAATCAGAACCCAAACA	GGGG**ACCACTTTGTACAAGAAAGCTGGGT**CGTAAGCCACGACCCCAGTG
LDAP3a	GGGG**ACAAGTTTGTACAAAAAAGCAGGCT**TAATGGCTGAAAATGATGTCAACAT	GGGG**ACCACTTTGTACAAGAAAGCTGGGT**CCTGGAAACCAATGGGGCTGATTCA
LDAP3b	GGGG**ACAAGTTTGTACAAAAAAGCAGGCT**TAATGGCTGAAAATGATGTTAACAT	GGGG**ACCACTTTGTACAAGAAAGCTGGGT**CGCTGGAAATCAAGGGGGTTGAT
